# Cell cycle–dependent translation-mediated turnover of the long noncoding RNA *Malat1*

**DOI:** 10.1083/jcb.202512059

**Published:** 2026-08-25

**Authors:** Leah M. Plasek-Hegde, Yeolhoe Kim, Rahul V. Gupta, Emily A. Dangelmaier, Sigrid Nachtergaele, Nadya Dimitrova

**Affiliations:** 1 Department of Molecular, Cellular, and Developmental Biology, Yale University, New Haven, CT, USA

## Abstract

Increased abundance of the nuclear long noncoding RNA (lncRNA) *Malat1* drives metastatic progression and is a strong predictor of poor patient prognosis. Although the mechanism that stabilizes *Malat1* through processing of its 3′ terminus is well-characterized, the pathways governing its turnover remain poorly understood. Here, we show that upon exit from mitosis, *Malat1* localizes to the cytoplasm, where it is degraded during early G1, resetting its abundance at the start of each cell cycle. Mechanistically, we demonstrate that *Malat1* turnover is mediated by a translation- and Smg1-dependent decay pathway and triggered by redundant elements. Importantly, failure to reset *Malat1* levels in early G1, due to decay inhibition or in the absence of progression through mitosis, results in *Malat1* accumulation. These findings uncover a cell cycle–dependent mechanism that harnesses the translation machinery to regulate *Malat1* abundance and identify cancer cell dormancy as a potential mechanism underlying the widespread overexpression of *Malat1* in cancer.

## Teaser

Plasek-Hedge et al. show that *Malat1*, a lncRNA thought to be exclusively nuclear, highly abundant, and very stable, in fact localizes to the cytoplasm in early G1, where it is degraded at the onset of each cell cycle by a translation-mediated decay pathway.

## Introduction

Widespread transcription of long noncoding RNAs (lncRNAs) is a defining feature of mammalian genomes (Carninci et al., 2005). Over the past two decades, hundreds of lncRNAs spanning a broad range of expression levels and subcellular localization patterns have been identified (Mattick et al., 2023). Although the physiological functions and mechanisms of most lncRNAs remain incompletely understood, a subset distinguished by evolutionary sequence conservation, high expression levels, and defined subcellular localization patterns has emerged as critical regulators of diverse biological processes (Winkler and Dimitrova, 2021). Among these, metastasis-associated lung adenocarcinoma transcript 1 (*Malat1*) has attracted significant attention due to its strong evolutionary conservation and enrichment in nuclear speckles (Hutchinson et al., 2007).

*Malat1* was originally identified as a transcript overexpressed in metastatic lung adenocarcinoma, where elevated expression was strongly associated with poor patient prognosis (Ji et al., 2003; Muller-Tidow et al., 2004). Subsequent studies in murine cancer models demonstrated that increased *Malat1* abundance is not merely correlated with aggressive disease but is sufficient to drive metastasis through epigenetic reprogramming of tumor cells and remodeling of the tumor microenvironment (Arun et al., 2016; Gutschner et al., 2013; Martinez-Terroba et al., 2024). These findings established aberrant upregulation of *Malat1* as a potent oncogenic driver of tumor progression.

Interestingly, *Malat1* is ubiquitously and highly expressed in both normal and transformed cells (Arun et al., 2020). Its exceptionally high abundance reflects both robust transcription and a long transcript half-life. Transcript stability is conferred by an unconventional RNase P–dependent 3′-end processing mechanism that cleaves nascent *Malat1* to generate a highly conserved expression and nuclear retention element (ENE) triple-helix structure, which protects the 3′ terminus from exonucleolytic degradation (Brown et al., 2014; Wilusz et al., 2008; Wilusz et al.,2012).

Although the mechanism that confers stability to *Malat1* has been extensively characterized, the pathways responsible for the turnover of ENE-stabilized *Malat1* remain poorly understood. Several studies have suggested that *Malat1* is subject to miRNA-mediated regulation (Leucci et al., 2013; Macias et al., 2012) or interacts with components of the nonsense-mediated decay (NMD) and Staufen-mediated decay (SMD) pathways (Xiao et al., 2024; Zund et al., 2013). However, these observations have been difficult to reconcile with the predominantly nuclear localization of *Malat1*, as these pathways function primarily in the cytoplasm. More recently, studies have identified RNA degradation pathways that are antagonized by the *Malat1* ENE (Che et al., 2025), yet the molecular pathways responsible for the turnover of ENE-protected *Malat1* have remained elusive.

To elucidate the mechanisms that regulate *Malat1* levels and may contribute to its dysregulation in cancer, we investigated *Malat1* abundance and subcellular localization during the cell cycle. Our findings reveal an unexpected cell cycle–coordinated mechanism for the turnover of mitotically inherited *Malat1* and explain the frequent dysregulation of its abundance in cancer.

## Results and discussion

### *Malat1* abundance correlates negatively with proliferation rate

**Figure S1.**
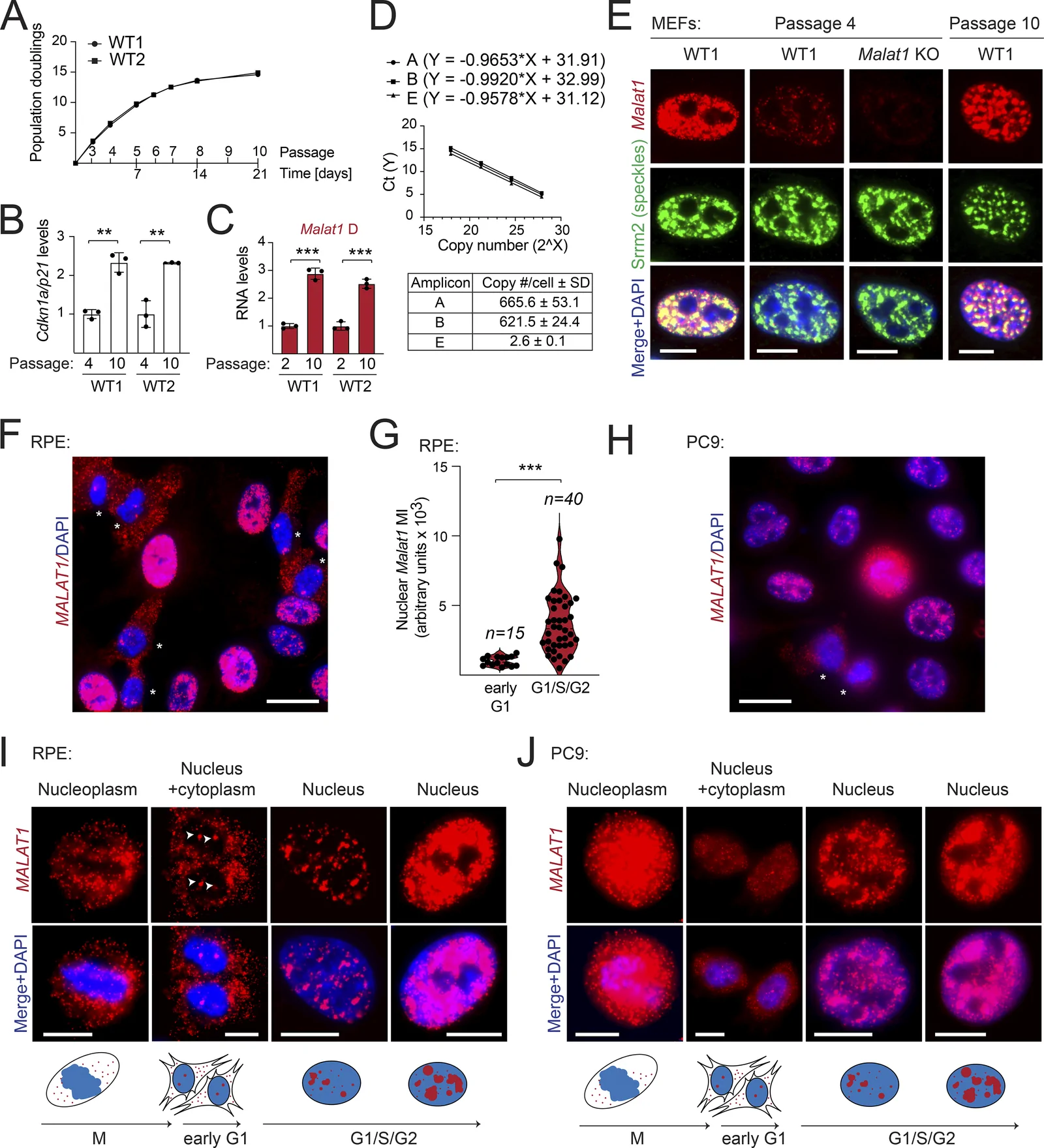
***Malat1/MALAT1* abundance and localization in murine and human cells, related to Figs. 1 and 2. (A)** Growth curve of two independent primary WT MEF lines, WT1 and WT2, over 10 passages. **(B)** qRT-PCR analysis of normalized *Cdkn1a/p21* RNA levels in cells from A at indicated passages. **(C)** qRT-PCR analysis of normalized *Malat1* RNA levels in cells from A at indicated passages, using a primer set specific to region D, excludes production of truncated *Malat1*. **(D)** Top, standard curve for copy number calculation using dsDNA templates, generated from genomic DNA with *Malat1* primer sets A, B, and E (Fig. 1 A); Bottom, number of copies per cell in WT1 MEFs at passage 2, detected with the indicated primer set. Unprocessed *Malat1*, detected with primer set E, represents < 1/200th (0.5%) of total *Malat1*, detected with primer sets A and B. **(E)** smRNA-FISH/IF detection of *Malat1* (red) and nuclear speckle marker, Srrm2 (green), in WT1 and KO MEFs at indicated passages. DAPI, DNA (blue). Scale bar: 10 μM. Representative images illustrate differences in *Malat1* abundance between passages, independent of speckles. **(F)** smRNA-FISH detection of *MALAT1* (red) in RPE cells. DAPI, DNA (blue). Scale bar: 20 μM. * indicate cells in early G1 with cytoplasmic *MALAT1* signal. **(G)** Quantification of nuclear *MALAT1* mean intensity (MI), measured in a.u., in cells with (early G1) and without (G1/S/G2) cytoplasmic *Malat1* in images from (F). **(H)** smRNA-FISH detection of *MALAT1* (red) in PC9 cells. DAPI, DNA (blue). Scale bar: 20 μM. * indicate cells in early G1 with cytoplasmic *MALAT1* signal. **(I and J)** Top, smRNA-FISH visualization of *MALAT1* (red) across different cell cycle stages in RPE (I) and PC9 (J) cells. DAPI, DNA (blue). Scale bar: 10 μM. Bottom, schematic of representative *MALAT1*/DAPI patterns in mitosis (M), early G1, and G1/S/G2 cell cycle stages. In this figure, bar graphs show individual values and mean ± SD of *n* = 3 technical replicates (B and C). *n* indicates the number of quantified nuclei represented in violin plots (G). Unpaired *t* test (B, C, and G), **P < 0.01; ***P < 0.001.

**Figure 1.**
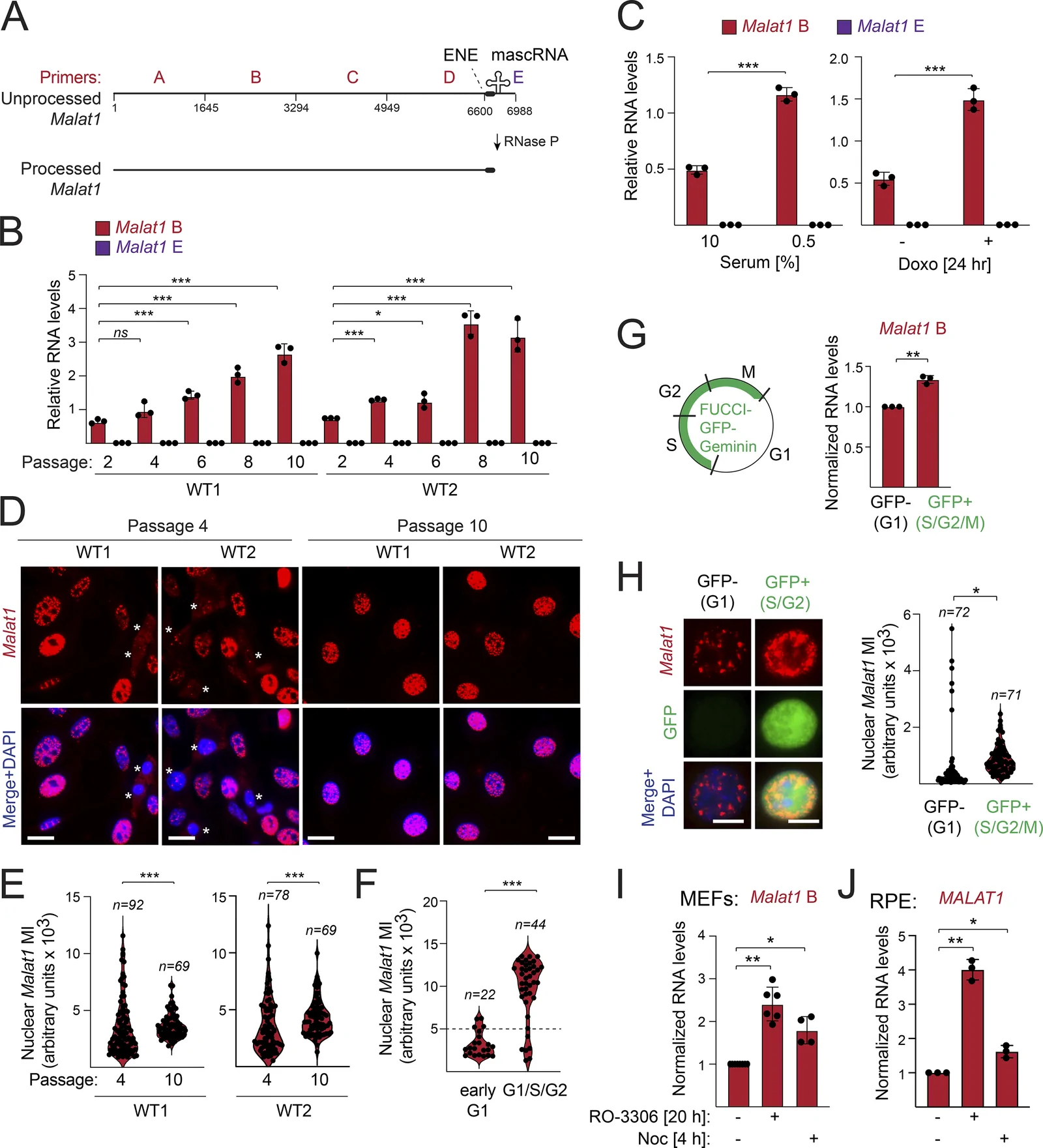
**Cell cycle–dependent regulation of *Malat1* abundance. (A)** Schematic of unprocessed and processed *Malat1* transcripts, highlighting the location of primers in regions A–E and the RNase P processing event, which removes the mascRNA-containing region E to generate the ENE terminus. **(B)** qRT-PCR detection of total and unprocessed *Malat1* RNA levels, detected with primer sets specific to regions B and E, relative to *Gapdh* in two independent primary WT MEF lines, WT1 and WT2, at indicated passages. **(C)** qRT-PCR detection of total and unprocessed *Malat1* RNA levels, detected with primer sets specific to regions B and E, relative to *Gapdh* in left, primary WT1 grown in 10% serum or upon starvation in 0.5% serum or right, primary WT1 untreated or treated with 0.5 μM doxorubicin (Doxo) for 24 h. **(D)** Panoramic images of smRNA-FISH detection of *Malat1* (red) in WT1 and WT2 MEFs from B at passage 4 and 10. DAPI, DNA (blue). * indicate cells in early G1 with cytoplasmic *Malat1* signal. Scale bar: 20 μM. **(E)** Violin plot of nuclear *Malat1* mean intensity (MI), measured in a.u., in WT1 and WT2 cells at indicated passages, quantified in images from D. **(F)** Violin plot of nuclear *Malat1* MI, measured in a.u., in WT1 cells at passage 4 that are positive (early G1) or negative (G1/S/G2) for cytoplasmic *Malat1* signal, quantified in images from D. **(G)** Left, schematic of FUCCI system; right, qRT-PCR analysis of normalized *Malat1* levels in GFP-negative (GFP-, G1) and GFP-positive (GFP+, S/G2/M) FUCCI-labeled cells sorted by FACS. **(H)** Left, co-detection of *Malat1* by smRNA-FISH (red) and GFP (green) in FUCCI-labeled cells. DAPI, DNA (blue). Right, violin plot of nuclear *Malat1* MI, measured in a.u., in GFP- or GFP+ cells. Scale bar: 10 μM. **(I)** qRT-PCR analysis of normalized *Malat1* levels in p53-deficient MEFs, treated with 8 μM RO-3306 or 50 ng/ml Noc, as indicated. **(J)** qRT-PCR analysis of normalized *MALAT1* levels in RPE cells treated with 8 μM RO-3306 or 50 ng/ml Noc, as indicated. In this figure, bar graphs show individual values and mean ± SD of *n* ≥ 3 biological (G, I, and J) and technical (B and C) replicates. *n* indicates the number of nuclei represented in violin plots (E, F, and H). Paired *t* test (G, I, and J), unpaired *t* test (B, C, F, G, and H), and sample variance (E); *P < 0.05; **P < 0.01; ***P < 0.001; *ns*, not significant.

We hypothesized that the frequent overexpression of *Malat1* in cancer may be linked to a common cancer cell feature, such as high proliferation rate. To test this, we compared *Malat1* levels in two independent early-passage primary WT E13.5 mouse embryonic fibroblast (MEF) lines (WT1 and WT2), which are highly proliferative, to late-passage MEFs, which are largely senescent (Fig. S1, A and B). Unprocessed and total *Malat1* were measured using primer sets specific to the 3′ (E) and 5′ (A–D) regions of the transcript, respectively (Fig. 1 A). Contrary to our hypothesis, we observed that the levels of *Malat1* progressively rose from passage 2 to 10 (Fig. 1 B and Fig. S1 C). This increase was predominantly due to accumulation of processed *Malat1*, as the levels of unprocessed *Malat1* accounted for <0.5% of total *Malat1* (Fig. S1 D). We also observed that processed *Malat1* levels increased approximately two to threefold in response to serum deprivation and upon acute exposure to the DNA damage-inducing agent, doxorubicin, in primary WT MEFs (Fig. 1 C). We concluded that *Malat1* abundance is inversely correlated to proliferation status.

### Cell cycle–dependent regulation of *Malat1* abundance

Visualization of *Malat1* by single-molecule RNA FISH (smRNA-FISH) (Raj et al., 2008) in WT1 and WT2 MEFs at passage 10 revealed uniform intercellular nuclear staining that overlapped with the nuclear speckle marker, Srrm2, detected by immunofluorescence (IF) (Fig. 1, D and E; and Fig. S1 E). In contrast, primary WT MEFs at passage 4 displayed markedly greater cell-to-cell variation in nuclear *Malat1* pattern and intensity, independent of speckles (Fig. 1, D and E; and Fig. S1 E). In particular, early G1 cells—identified by the close proximity of the daughter nuclei and the presence of cytoplasmic *Malat1*, previously observed in cells exiting mitosis (Blower et al., 2023; Hutchinson et al., 2007)—consistently exhibited lower nuclear *Malat1* signal than cells at other stages of interphase (Fig. 1, D and F). This observation suggested that increased heterogeneity of nuclear *Malat1* staining in proliferating cells may reflect differences in cell cycle stage distribution.

To independently assess *Malat1* abundance across the cell cycle, we introduced the GFP-tagged Geminin component of the fluorescent ubiquitination-based cell cycle indicator (FUCCI) system, which is stabilized in the S/G2/M stages of the cell cycle (Fig. 1 G) (Sakaue-Sawano et al., 2008). Consistent with cell cycle–dependent regulation, qRT-PCR and smRNA-FISH both showed significantly lower *Malat1* abundance in GFP-negative G1 cells than in GFP-positive S/G2/M cells (Fig. 1, G and H). Conversely, arrest in late G2 or mitosis using the CDK1 inhibitor, RO-3306, or the microtubule polymerization inhibitor, Nocodazole (Noc), respectively, increased *Malat1* levels approximately two to threefold relative to asynchronous cells (Fig. 1 I). Importantly, similar cell cycle–dependent pattern was observed for human *MALAT1* in retinal pigment epithelium (RPE) and patient-derived EGFR-mutant lung adenocarcinoma (PC9) cells (Fig. S1, F–H and Fig. 1 J). Together, these findings demonstrate that the abundance of murine *Malat1* and human *MALAT1* fluctuates during the cell cycle, reaching its lowest levels in G1 and highest levels in G2/M.

### *Malat1* is degraded in the postmitotic early G1 cytoplasm

**Figure 2.**
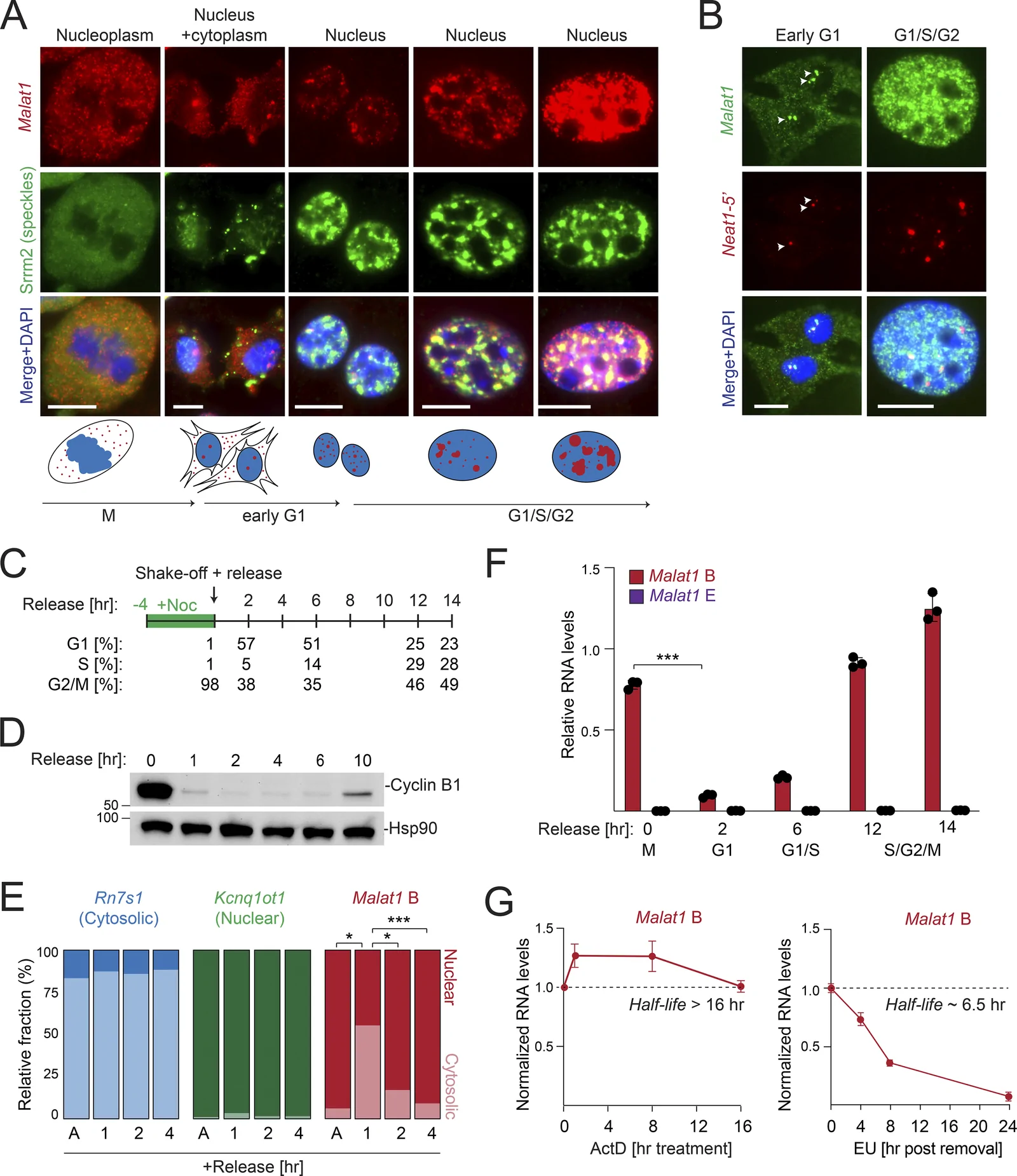
**Degradation of mitotically inherited cytoplasmic *Malat1* in early G1. (A)** Top, representative images of smRNA-FISH/IF detection of *Malat1* (red) and nuclear speckle marker, Srrm2 (green), in WT1 primary MEFs at passage 4. DAPI, DNA (blue). Scale bar: 10 μM. Bottom, schematic of representative *Malat1*/DAPI patterns in mitosis (M), early G1, or G1/S/G2 cell cycle stages. **(B)** smRNA-FISH co-detection of *Malat1* (green) and *Neat1-5*′ (red) in cells from A with presence (early G1) or absence (G1/S/G2) of cytoplasmic *Malat1* staining. DNA, DAPI (blue). Arrowheads indicate co-localization of *Malat1* and *Neat1-5′* signals at sites of transcription in early G1. Scale bar: 10 μM. **(C)** Top, schematic of mitosis synchronization strategy of p53-deficient MEFs by treatment with 50 ng/ml Noc for 4 h, followed by manual shake-off of mitotic cells, and release into Noc-free media for the indicated time. Bottom, cell cycle stage distribution by FACS of cells collected at indicated time points post mitotic release (pmr). **(D)** Immunoblot detection of mitotic marker cyclin B1 in whole-cell extract isolated from cells, collected at indicated time points pmr, as described in C. Hsp90, loading control. Molecular weight markers, kDa. **(E)** qRT-PCR analysis of the relative abundance of *Rn7s1* (cytosolic marker), *Kcnq1ot1* (nuclear marker), and *Malat1* in nuclear and cytosolic fractions from p53-deficient MEFs, harvested asynchronously (A) or at indicated time points pmr, as described in C. Bar graphs show the mean of *n* = 3 biological replicates. Paired *t* test, *P < 0.05; ***P < 0.001. **(F)** qRT-PCR detection of total and unprocessed relative *Malat1* RNA levels, detected with primer sets specific to regions B and E, relative to *Gapdh* in p53-deficient MEFs, collected at indicated time points pmr, as described in C. Bar graph shows mean ± SD of *n* = 3 technical replicates. Paired *t* test, ***P < 0.001. Data were confirmed in a biological replicate. **(G)** qRT-PCR detection of normalized *Malat1* levels in left, p53-deficient MEFs treated with 5 μg/ml ActD for the indicated time. Half-life could not be determined due to lack of observable decay over the experimental time course; and right, p53-deficient MEFs pulsed for 1 h with 0.5 mM 5-EU and released in EU-free media for the indicated time. Half-life was determined using nonlinear regression of one-phase decay. Graph shows mean ± SD of *n* = 3 technical replicates. Source data are available for this figure: SourceData F2.

To determine why *Malat1* abundance is lower in G1 than G2/M cell cycle stages, we examined its localization and levels during the M-to-G1 transition by smRNA-FISH. Consistent with previous reports, we observed that *Malat1/MALAT1* dissociates from chromatin during mitosis and disperses throughout the nucleoplasm in MEFs, RPE, and PC9 cells (Fig. 2 A; and Fig. S1, I and J) (Blower et al., 2023; Sharp et al., 2020). Also consistent with prior reports, upon mitotic exit, inherited *Malat1/MALAT1* was found to remain in the cytoplasm of daughter cells as hundreds of discrete foci corresponding to individual RNA molecules (Fig. 2 A; and Fig. S1, I and J) (Blower et al., 2023; Hutchinson et al., 2007). In contrast, we observed that the nuclei of early G1 cells contain, on average, only 4.1 *Malat1* foci (Fig. 2 A, arrows, and Fig. S1, I and J), of which 67% co-colocalize with *Neat1*, a lncRNA transcribed in genomic proximity to *Malat1*, indicating that they primarily represent sites of nascent *Malat1* transcription (Fig. 2 B). Thus, in early G1 cells, which comprise 8.5 ± 4.3% and 12.5 ± 2.6% of asynchronous MEFs and RPE populations, respectively, mitotically inherited *Malat1* remains in the cytoplasm, while newly synthesized *Malat1* accumulates in the nucleus. As cells progress through interphase, nuclear *Malat1* signal was observed to increase and adopt its characteristic speckle pattern, while cytoplasmic *Malat1* foci were no longer detectable (Fig. 1 D, Fig. 2 A, and Fig. S1, F–J).

We confirmed these observations using cell cycle synchronization. p53-deficient MEFs were arrested in mitosis with Noc, collected by mitotic shake-off, and synchronously released into the next cell cycle (Fig. 2, C and D). qRT-PCR analysis of subcellular fractions at 1 h postmitotic release (pmr) revealed significant enrichment of cytoplasmic *Malat1* relative to asynchronous cells, followed by a significant depletion at 2 and 4 h pmr (Fig. 2 E). These results confirmed that mitotically inherited *Malat1* transiently localizes to the cytoplasm in early G1.

To determine the fate of cytoplasmic *Malat1*, we analyzed *Malat1* levels by qRT-PCR at different time points pmr. Strikingly, we observed that *Malat1* levels dropped by over 80% at 2 h pmr compared with M phase–arrested cells, indicating that the majority of inherited *Malat1* is degraded in early G1 (Fig. 2 F). The sharp reduction of *Malat1* levels was followed by gradual accumulation of *Malat1* at consecutive time points pmr, consistent with new transcription as cells progressed through the cell cycle (Fig. 2 F). Together, these findings demonstrated that mitotically inherited *Malat1* is degraded in the postmitotic cytoplasm, explaining its lower abundance in G1 compared with G2/M.

Moreover, *Malat1* turnover appeared to be limited to early G1, as *Malat1* levels did not significantly change over a 16-h treatment with the transcription inhibitor actinomycin D (ActD), which suppresses mitotic progression (Fig. 2 G). In contrast, when we used a 5-ethynyluridine (EU) pulse to label newly synthesized RNA in the absence of cell cycle perturbations, we determined that the half-life of the *Malat1* transcript is 6.5 h, consistent with the cell cycle length in p53-deficient MEFs (Fig. 2 G).

### Translation- and Smg1-dependent decay of cytoplasmic *Malat1*

**Figure 3.**
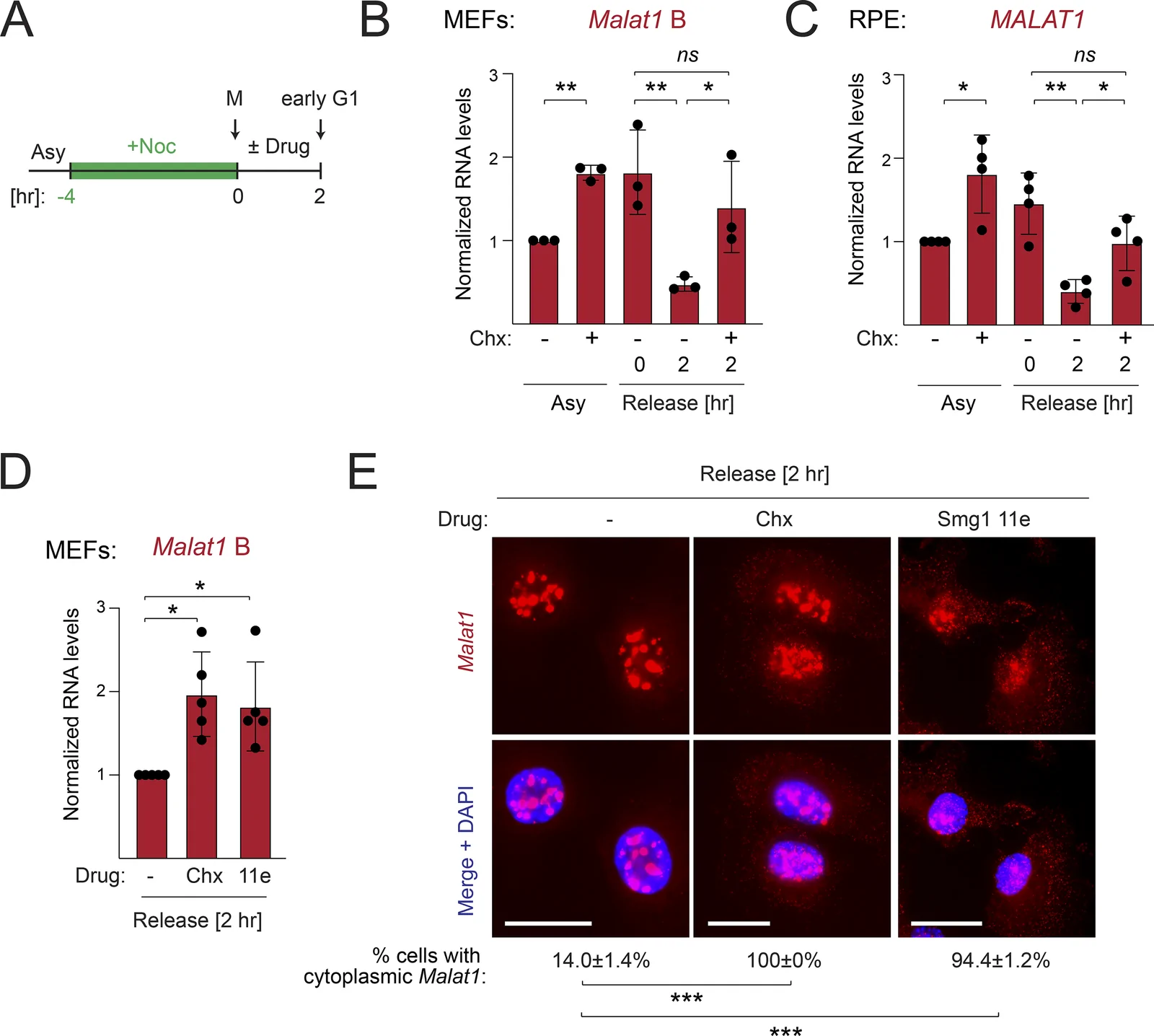
**Translation- and Smg1-mediated decay of cytoplasmic *Malat1*. (A)** Schematic of mitotic arrest and release experimental setup. Asynchronous cells (Asy) were treated for 4 h with 50 ng/ml Noc, followed by shake-off and release of mitotic cells in Noc-free media for 2 h in the absence of presence of drug. Cells collected by shake-off at 0 h after release represent mitotic (M) cells, while cells harvested at 2 h after release represent early G1 cells. **(B and C)** qRT-PCR analysis of normalized *Malat1* (B) or *MALAT1* (C) RNA levels in p53-deficient MEFs or RPE cells, respectively, harvested at indicated time points postmitotic arrest with 50 ng/ml Noc in the absence or presence of 50 μg/ml Chx, as described in A. **(D)** qRT-PCR analysis of normalized *Malat1* RNA levels in p53-deficient MEFs, harvested at 2 h pmr in the presence of no drug, 50 μg/ml Chx, or 5 μM hSmg1 inhibitor 11e (11e), as described in A. **(E)** smRNA-FISH detection of *Malat1* (red) in cells fixed at 2 h pmr in the presence of no drug, 50 μg/ml Chx, or 5 μM hSmg1 inhibitor 11e (11e), as described in A. DNA, DAPI (blue). Scale bar: 20 μM. Numbers below indicate the fraction of cells with 50 or more cytoplasmic *Malat1* foci in three biological replicates, with *n* > 50 cells scored per replicate. In this figure, bar graphs (B–D) show individual values and mean ± SD of *n* ≥ 3 biological replicates. Paired *t* test (B–E), *P < 0.05; **P < 0.01; ***P < 0.001; *ns*, not significant.

**Figure S2.**
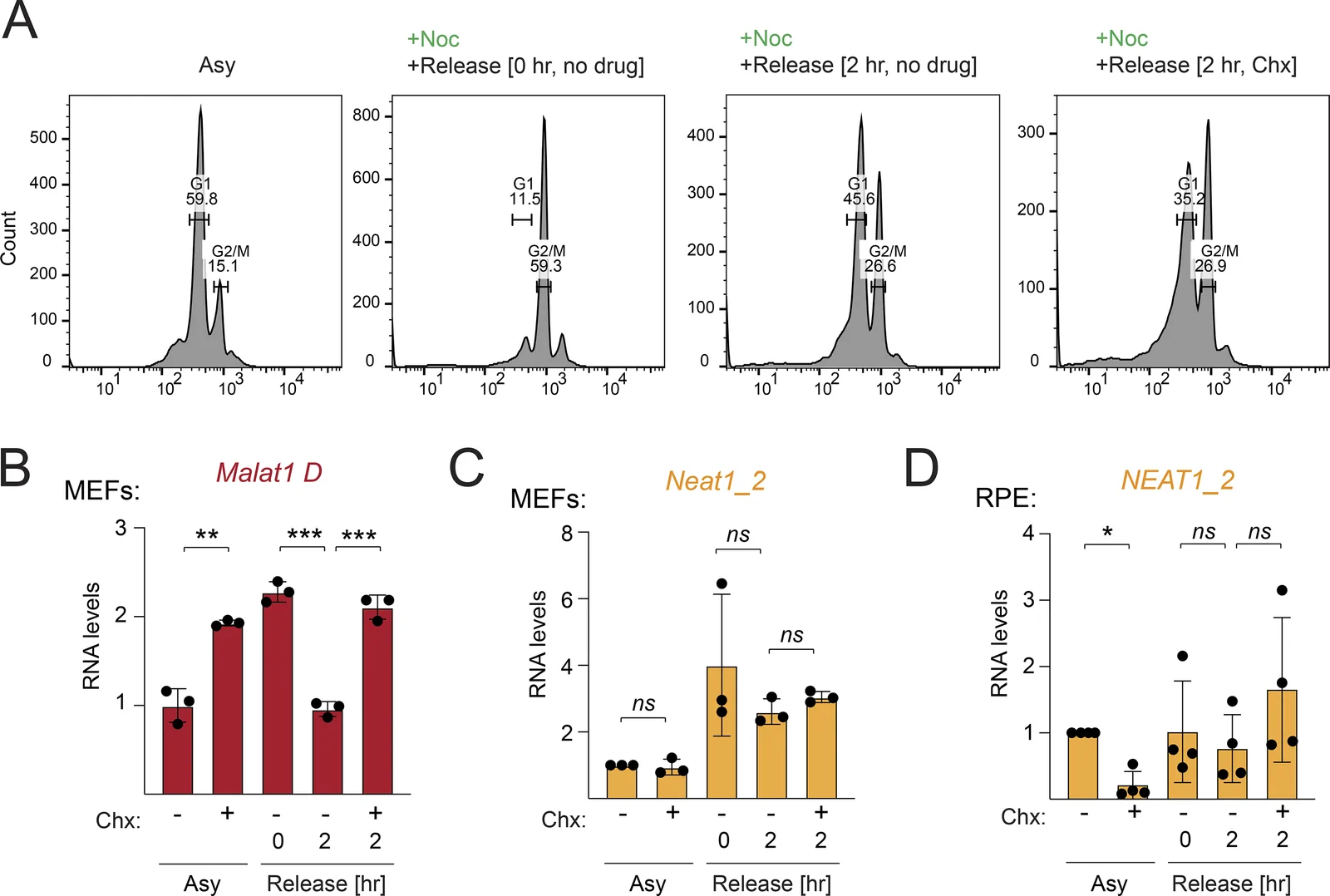
**Supporting data for translation inhibition studies, related to Fig. 3. (A)** FACS analysis of p53-deficient MEFs treated as described in Fig. 3 A. Data confirm enrichment of G2/M cells in samples treated for 4 h with 50 ng/ml Noc and entry into G1 of cells released from Noc in the absence or presence of 50 μg/ml Chx for 2 h. **(B)** qRT-PCR analysis of normalized *Malat1* RNA levels in samples, described in Fig. 3 B, using a primer set specific to region D, excludes production of truncated *Malat1*. **(C and D)** qRT-PCR analysis of normalized *Neat1_2* (C) or *NEAT1_2* (D) RNA levels in murine and human cells, described in Fig. 3, B and C, respectively, shows that *Neat1_2* and *NEAT1_2* are not subject to the same translation-dependent regulatory mechanism as *Malat1/MALAT1*. In this figure, bar graphs show individual values and mean ± SD of *n* ≥ 3 technical (B) and biological (C and D) replicates. Unpaired *t* test (B) and paired *t* test (C and D), *P < 0.05; **P < 0.01; ***P < 0.001; *ns* not significant.

As *MALAT1* has been reported to associate with translating ribosomes (Wilusz et al., 2012) and interact with components of the NMD and SMD pathways (Xiao et al., 2024; Zund et al., 2013), we asked whether *Malat1* is degraded by a translation-dependent cytoplasmic decay pathway in early G1. To test this, we treated asynchronous cells or cells released from mitotic arrest with the translation inhibitor cycloheximide (Chx) for 2 h (Fig. 3 A). FACS analysis confirmed that cells progressed from mitosis into G1 despite inhibition of new protein synthesis, consistent with prior reports (Fig. S2 A) (Verbin and Farber, 1967). Importantly, Chx treatment increased *Malat1/MALAT1* levels ∼1.8-fold in asynchronous MEFs and RPE cells (Fig. 3, B and C; and Fig. S2 B) and rescued postmitotic *Malat1/MALAT1* degradation following mitotic release (Fig. 3, B and C; and Fig. S2 B). These findings revealed a translation-dependent mechanism for *Malat1/MALAT1* degradation in early G1. In contrast, *Neat1_2/NEAT1_2*, the other mammalian lncRNA terminating in an ENE protective structure, did not undergo significant degradation in early G1, suggesting that this mechanism is specific to *Malat1/MALAT1* (Fig. S2, C and D).

Treatment with Smg1 11e, a selective inhibitor of the shared NMD/SMD kinase Smg1 (Gopalsamy et al., 2012), similarly increased *Malat1* abundance approximately twofold at 2 h pmr, supporting a role for NMD or SMD in *Malat1* degradation (Fig. 3 D). Moreover, at 2 h pmr, cytoplasmic *Malat1* was detected in 100 ± 0% and 94.4 ± 1.2% of MEFs, treated with Chx or Smg1 11e, respectively, compared with 14.0 ± 1.4% of untreated cells (Fig. 3 E). Together, these findings demonstrate that cytoplasmic *Malat1* is degraded in early G1 through a translation- and Smg1-dependent pathway.

### *Malat1* degradation is triggered by redundant elements

**Figure S3.**
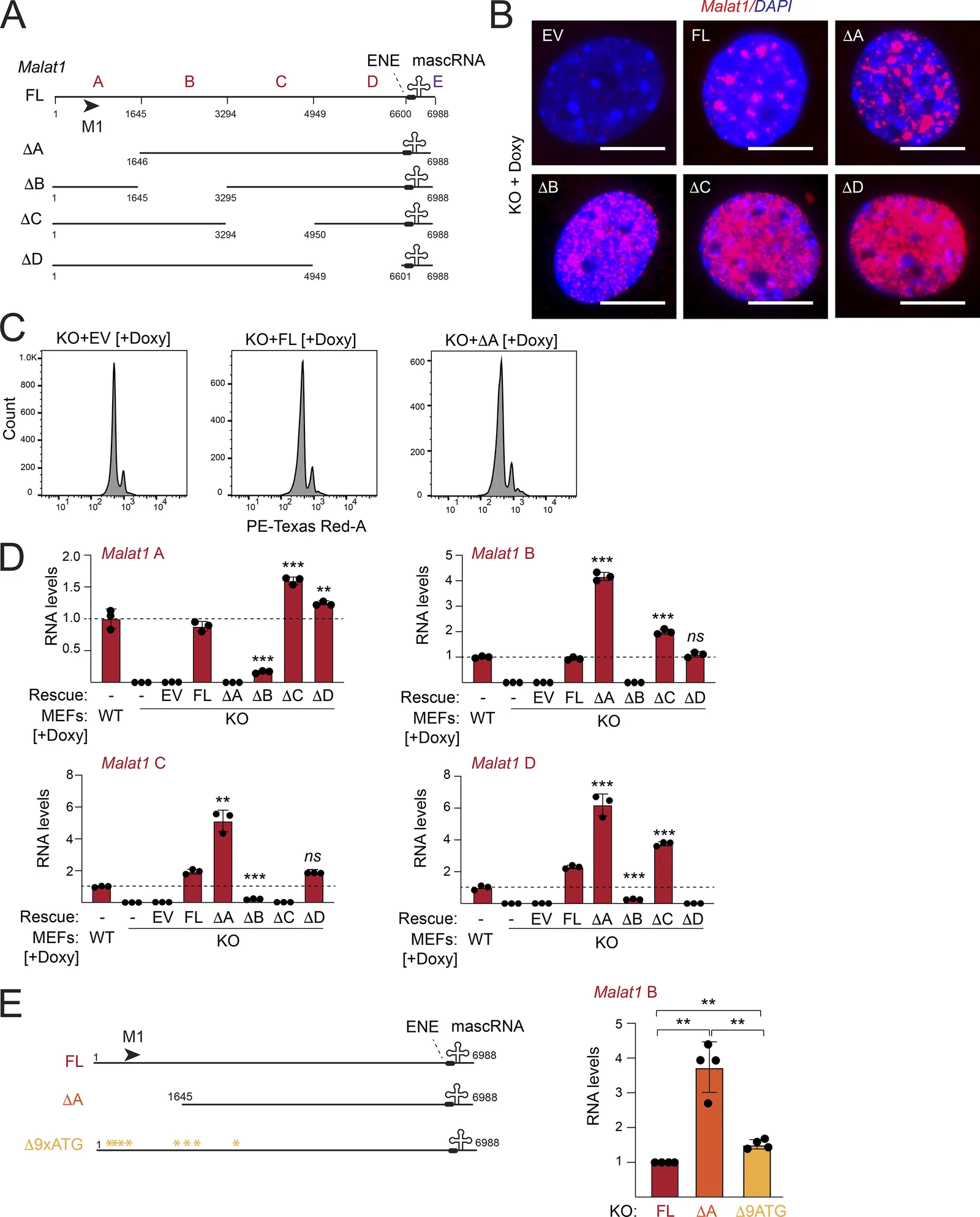
**Characterization of *Malat1* WT and mutant complementation constructs, related to Fig. 4. (A)** Schematic of Doxy-regulated FL and region A–D deletion *Malat1* constructs (ΔA–ΔD), expressed downstream of a TRE cassette in a PiggyBac (PB) vector, co-expressing rtTA (PB-TRE-rtTA). All constructs contain region E, required for the formation of the stabilizing ENE. **(B)** smRNA-FISH detection of *Malat1* (red) in *Malat1* KO MEFs, complemented with empty vector (EV), FL, or ΔA–ΔD *Malat1*, cultured in the presence of 1 μg/ml Doxy for 3 days. DAPI, DNA (blue). Scale bar: 10 μM. Representative images show that B–D region deletions affect speckle localization, while region A deletion does not. **(C)** FACS analysis of indicated cells, cultured in the presence of 1 μg/ml Doxy for 3 days, shows comparable cell cycle distributions in EV, FL, and ΔA-expressing KO cells. **(D)** qRT-PCR analysis of normalized *Malat1* levels using primers located in indicated regions A–D, in WT, KO, and KO MEFs, complemented with EV, FL, or ΔA-ΔD *Malat1*, cultured in the presence of 1 μg/ml Doxy for 3 days. Bar graphs show individual values and mean ± SD of *n* = 3 technical replicates. Unpaired *t* test was used to perform pairwise comparisons between ΔA–ΔD and FL samples. **P < 0.01; ***P < 0.001; *ns*, not significant. Data indicate that ΔA and ΔC are significantly more abundant compared with FL. **(E)** Left, schematic of FL, ΔA, and Δ9xATG *Malat1* constructs, highlighting the location of the nine mutated sORF ATGs, including M1. Right, qRT-PCR analysis of normalized *Malat1* levels in *Malat1* KO cells complemented with FL, ΔA, and Δ9xATG, cultured in the presence of 1 μg/ml Doxy for 3 days. Bar graph shows individual values and mean ± SD of *n* = 4 biological replicates. Paired *t* test, **P < 0.01.

To identify the region of *Malat1* that triggers decay, we complemented *Malat1* knockout (KO) MEFs, immortalized by p53 inactivation, with constructs expressing doxycycline (Doxy)-inducible empty vector (KO+EV), full-length *Malat1* (KO+FL), or *Malat1* deletion mutants lacking 1.6-kb regions A–D (KO+ΔA–D) (Fig. 1 A and Fig. S3 A). All *Malat1* constructs contained region E to enable posttranscriptional processing of *Malat1* into an ENE-terminating transcript (Fig. 1 A and Fig. S3 A). Analyses of KO+FL and KO+ΔA–D cells revealed that deletion of region A did not affect speckle localization or cell cycle progression (Fig. S3, B and C) but led to significantly increased abundance of ΔA relative to endogenous and FL *Malat1*, indicating a role for region A in *Malat1* destabilization (Fig. S3 D). Notably, region A has been reported to strongly associate with translating ribosomes and encode a small ORF (sORF), making it a candidate for an NMD trigger (Fig. S3 A) (Wilusz et al., 2012; Xiao et al., 2024).

**Figure 4.**
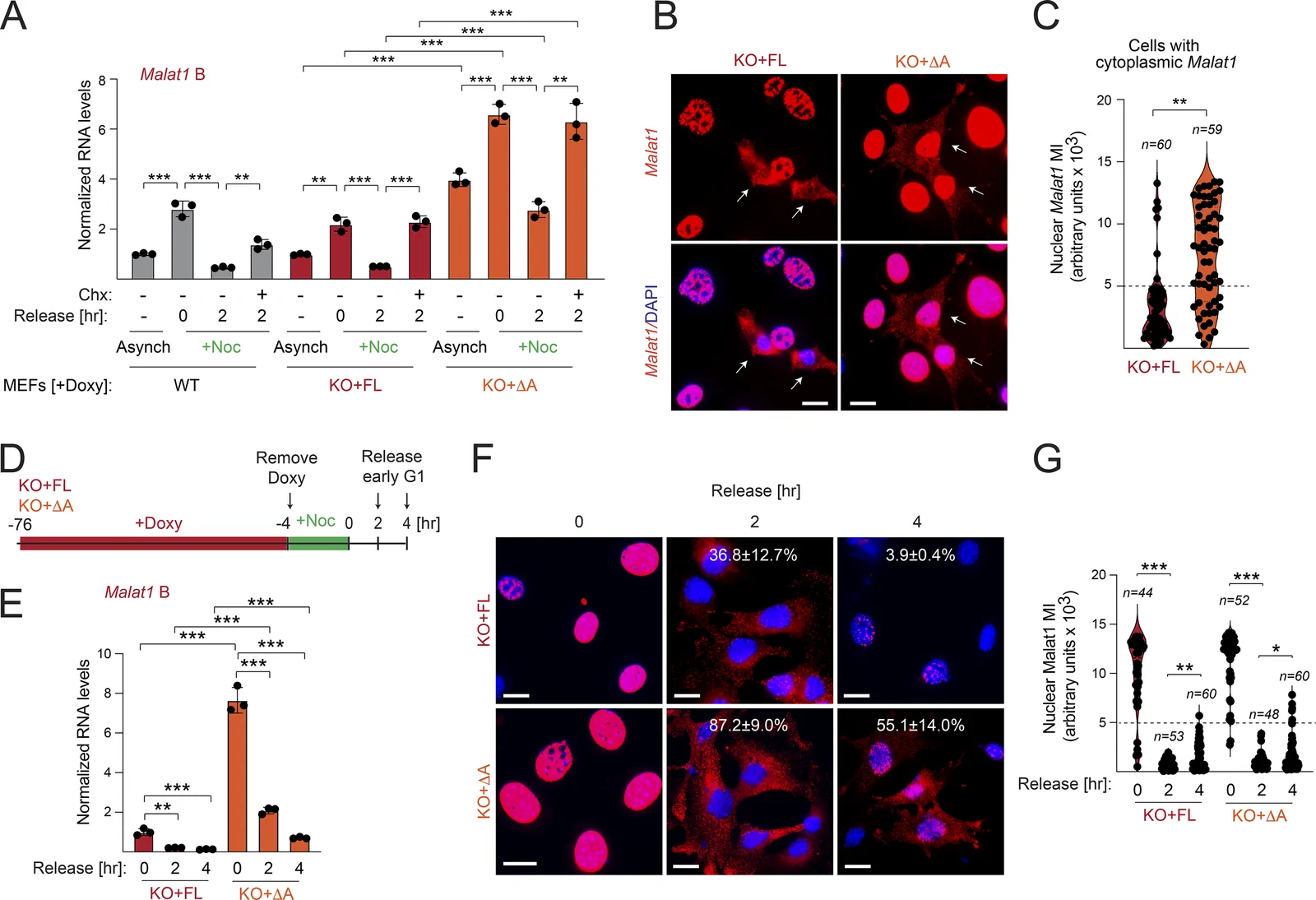
**Redundant elements in *Malat1* trigger postmitotic degradation. (A)** qRT-PCR analysis of normalized *Malat1* RNA levels in p53-deficient *Malat1* WT and KO MEFs, rescued with Doxy-inducible FL (KO+FL) and ΔA (KO+ΔA) *Malat1* constructs, cultured for 3 days in the presence of 1 μg/ml Doxy and harvested asynchronous or at indicated time points after release from mitotic arrest with 50 ng/ml Noc in the absence or presence of 50 μg/ml Chx, as described in Fig. 3 A. **(B)** smRNA-FISH of *Malat1* (red) in asynchronous KO+FL and KO+ΔA cells, cultured in the presence of 1 μg/ml Doxy for 3 days. DAPI, DNA (blue). Arrows point to cells with cytoplasmic *Malat1* signal. Scale bar: 20 μM. **(C)** Violin plot of nuclear *Malat1* MI, measured in a.u., in KO+FL and KO+ΔA cells with cytoplasmic *Malat1*, quantified in images from B. **(D)** Schematic of the experiment, showing Doxy-dependent induction of FL and ΔA expression for 3 days, followed by removal of Doxy, mitotic synchronization, and release into G1 for indicated times. **(E)** qRT-PCR analysis of normalized *Malat1* RNA levels in KO+FL and KO+ΔA, collected at indicted time points, as described in D. **(F)** smRNA-FISH of *Malat1* (red) in KO+FL and KO+ΔA cells, fixed after 4 h of Noc treatment (0 h) or replated for indicated time following mitotic shake-off (2 and 4 h), as described in D. DNA, DAPI (blue). Scale bar: 20 μM. Note that, at 0 h, the mitotic cells were washed away during the fixation and staining procedure. Numbers indicate the fraction ± SD of cells with 50 or more cytoplasmic *Malat1* foci from three biological replicates, with *n* > 100 cells scored per replicate. **(G)** Violin plot of nuclear *Malat1* MI, measured in a.u., quantified in images from F. In this figure, bar graphs show mean ± SD of *n* = 3 technical replicates, validated in biological replicates (A and E). *n* indicates the number of nuclei represented in violin plots (C and G). Unpaired *t* test (A, C, E, and G), *P < 0.05; **P < 0.01; ***P < 0.001; *ns*, not significant.

We observed that ΔA transcripts were 5.5-fold more abundant than FL at 2 h pmr, indicating that ΔA is not degraded to the same extent as FL *Malat1* in early G1 (Fig. 4 A). However, we also observed that over 50% of ΔA was degraded at 2 h pmr relative to non-released ΔA-expressing cells and that this degradation was reversed by Chx, indicating that ΔA is susceptible to translation-dependent degradation (Fig. 4 A). We concluded that region A contributes to but is not required for *Malat1* degradation.

We also examined the relative contribution of sORFs in region A by generating a Δ9xATG *Malat1* mutant, lacking initiation codons of 9 sORFs, including previously described M1 sORF (Fig. S3 E) (Xiao et al., 2024). Analysis of asynchronous cells revealed that Δ9xATG *Malat1* was ∼1.5-fold more abundant compared with FL but significantly less abundant compared with ΔA (Fig. S3 E). This observation further supports the conclusion that degradation of *Malat1* is dependent on redundant elements, with elements in region A and the 9 sORFs being necessary, but not sufficient, for degradation.

### Cytoplasmic persistence and nuclear import of undegraded *Malat1*

Quantification of nuclear *Malat1* abundance in early G1 cells revealed significantly higher mean signal intensity in ΔA than FL-expressing KO cells (Fig. 4. B and C). We reasoned that this could reflect either prolonged cytoplasmic persistence of ΔA *Malat1* into later stages of the cell cycle or nuclear reimport of undegraded cytoplasmic transcripts. To distinguish between these possibilities, we transiently induced FL and ΔA *Malat1* expression with Doxy, then synchronized cells in mitosis, and released them into the next cell cycle in the absence of Doxy (Fig. 4 D). Consistent with degradation of mitotically inherited *Malat1* at the onset of each cell cycle, FL and ΔA *Malat1* levels were reduced by 80% at 2 h pmr relative to non-released cells, and this reduction persisted at 4 h pmr in the absence of new transcription (Fig. 4. E and F). Notably, ΔA *Malat1* remained ∼10-fold more abundant than FL at both time points, indicating incomplete degradation in the absence of region A, as reported above (Fig. 4, A and E). Quantification of the fraction of cells with cytoplasmic *Malat1* by smRNA-FISH revealed that this increase was partly due to prolonged cytoplasmic persistence of ΔA *Malat1* compared with FL (87.2 ± 9.0% vs. 36.8 ± 12.7% at 2 h pmr; 55.1 ± 14.0% vs. 3.9 ± 0.4 at 4 h pmr, Fig. 4 F).

Interestingly, we observed that nuclear *Malat1* signal increased between 2 and 4 h pmr in both FL- and ΔA-expressing cells (Fig. 4 G). Because no new *Malat1* transcription occurred during this period, this finding indicated that a fraction of cytoplasmic *Malat1* escapes degradation and is reimported into the nucleus. Together, these analyses show that incomplete degradation of *Malat1* in early G1 can result in both prolonged cytoplasmic persistence, particularly in ΔA mutants, and nuclear re-import of undegraded transcripts.

### *Malat1* accumulates in dormant cancer cell populations

**Figure 5.**
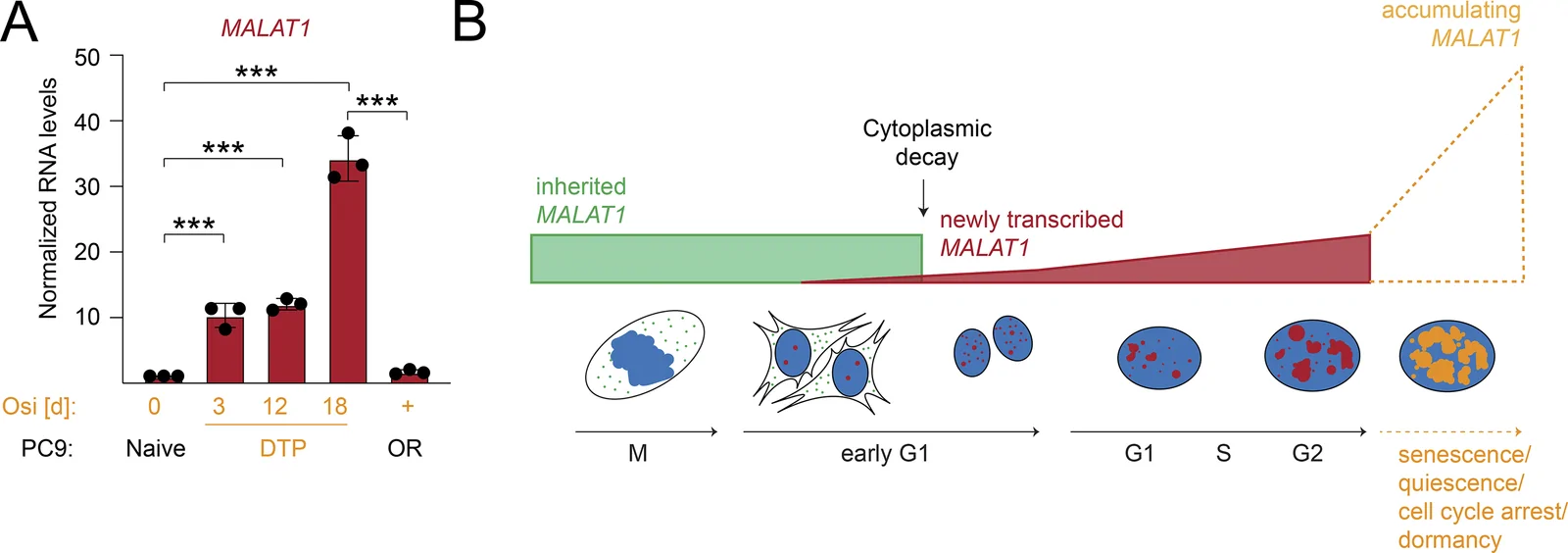
**Cell dormancy enables *MALAT1* overexpression in cancer. (A)** qRT-PCR analysis of normalized *MALAT1* RNA levels in PC9 cells treated with 100 nM Osi for the indicated days (d). OR, Osi resistant. OR cells are cultured in the presence of 100 nM Osi. Bar graph shows individual values and mean ± SD of *n* = 3 biological replicates, paired *t* test, ***P < 0.01. **(B)** Model for cell cycle–dependent regulation of *MALAT1* abundance, showing gradual accumulation of newly transcribed *MALAT1* (red) in early G1 to late G2 nuclei, followed by dissociation from chromatin in mitosis and decay of post-mitotically inherited *MALAT1* (green) in the cytoplasm. In the absence of passage through mitosis, continuously transcribed *MALAT1* (orange) accumulates in the nuclei of senescent, quiescent, cell cycle–arrested, or dormant cells.

Dormant cancer cell subpopulations that are transcriptionally active but non-proliferative are an established reservoir for metastasis, tumor relapse, and drug resistance (Klein, 2020; Russo et al., 2024). Because we determined that passage through mitosis is required for the cytoplasmic localization and early G1 degradation of *Malat1*, we wondered whether proliferative dormancy contributes to the overexpression of *Malat1* in cancer. We analyzed *MALAT1* abundance in EGFR-mutant PC9 cells treated with the tyrosine kinase inhibitor osimertinib (Osi), which enter a slow-cycling, drug-tolerant persister (DTP) state, where priming for drug resistance evolution occurs (Starble et al., 2025). Consistent with our model, *MALAT1* levels progressively rose in DTP cells but declined once cells acquired Osi resistance and resumed proliferation in the continued presence of Osi (Fig. 5 A). These findings indicate that reduced proliferation is a direct mechanism driving *MALAT1* overexpression.

Taken together, our work reveals a model for the dynamic regulation of *Malat1* abundance that also explains its dysregulation in cancer. Whereas previous studies focused on *Malat1* accumulation and stability in the interphase nucleus, we show that mitotically inherited *Malat1* localizes to the cytoplasm, where it is degraded in the first 2 h of G1, resetting its abundance at the onset of each cell cycle (Fig. 5 B). We further demonstrate that non-proliferative cell states, including quiescence, senescence, cell cycle arrest, and dormancy, accumulate high levels of *Malat1* because they do not progress through mitosis and therefore fail to execute this early G1 degradation program (Fig. 5 B). These findings raise the possibility that changes in proliferation state alone, without genetic or epigenetic rewiring, may contribute to the widespread overexpression of *Malat1* observed across cancers. Given that both *Malat1* overexpression (Arun et al., 2016; Gutschner et al., 2013; Martinez-Terroba et al., 2024) and tumor cell dormancy (Hinterleitner et al., 2026) promote metastatic progression, it will be important to determine whether they functionally converge.

Mechanistically, our findings reveal an unexpected twist on the central dogma, where the translation machinery is co-opted to mediate the turnover of one of the most abundant and stable mammalian lncRNAs. Our work clarifies that the previously reported ribosomal occupancy at the 5′ end of *Malat1* (Wilusz et al., 2012) likely reflects engagement with ribosomes in the postmitotic cytoplasm. Recent studies have also identified translated *Malat1* sORFs, including the neuron-specific 33-aa M1 peptide (Rai et al., 2026; Xiao et al., 2024). This could result from specialized trafficking of *Malat1* to the cytoplasm during neuronal interphase or from reduced postmitotic decay efficiency in neurons (Palou-Marquez and Supek, 2025), which prolongs cytoplasmic *Malat1* residence and enables M1 translation. These two models can be distinguished by examining whether quiescent neurons harbor cytoplasmic *Malat1* and express the M1 peptide.

Our work implicates an Smg1-dependent decay mechanism, such as NMD or SMD, in *Malat1* turnover. Indeed, we observed cytoplasmic retention of *Malat1* in over 90% of early G1 cells treated with an Smg1 inhibitor compared with 14% in control samples. How *Malat1* engages these decay mechanisms remains unclear. NMD has been proposed to act primarily through the “exon-junction complex (EJC) model,” where the translating ribosomes rely on the EJC to distinguish premature from normal termination codons (Kim et al., 2001; Le Hir et al., 2001; Lykke-Andersen et al., 2001). While *Malat1* is generally not considered to be spliced, a recent report has indicated that *Malat1* can be spliced in the absence of association with the splicing regulator U2AF2 (Grammatikakis et al., 2026, *Preprint*). It will be interesting to determine whether cytoplasmic *Malat1* is bound by U2AF2 in early G1. As an alternative, the “Faux 3′UTR model” has been proposed for NMD of unspliced mRNAs with unusually long unstructured 3′UTRs that limit productive interactions between the terminating ribosome at the PTC and the poly(A)-binding protein, PABPC1 (Amrani et al., 2004). In support of this model, processed *Malat1* is a 7-kb noncoding transcript that is unlikely to effectively recruit Pabpc1 as it lacks a canonical poly(A) tail. Additionally, *Malat1* was recently reported to co-localize with Staufen in neurons (Xiao et al., 2024), suggesting a potential role for the SMD pathway (Kim et al., 2005). Although region A and nine sORFs were found to contribute to *Malat1* destabilization, they were not required, suggesting redundant elements, such as additional sORFs or secondary structures. Indeed, *Malat1* contains 41 sORFs (>20 aa), 33 located outside region A, as well as multiple reported structured RNA elements (McCown et al., 2019; Monroy-Eklund et al., 2023; Xiao et al., 2026, *Preprint*). Lastly, our work does not exclude the complementary or redundant role of RNA interference (RNAi) in the regulation of cytoplasmic *Malat1* in early G1. Further work will define the relative contribution of various functional elements and mechanisms to the regulation of *Malat1* turnover.

## Materials and methods

### Cell lines and constructs

All cells were maintained at 37°C in a humidified incubator with 5% CO_2_. Primary *Malat1* WT and KO MEFs were isolated from E13.5 littermate embryos from timed matings of *Malat1* heterozygous mice (*Malat1*^*+/−*^), generously provided by Dr. David Spector (CSHL, Cold Spring Harbor, NY, USA) (Zhang et al., 2012). Primary MEF lines were established and passaged in DMEM (Gibco) supplemented with 15% fetal bovine serum, 50 U ml^−1^ penicillin-streptomycin, 2 mM L-glutamine, 0.1 mM nonessential aa, and 0.055 mM 2-mercaptoethanol. Experiments in primary MEFs were performed between passages 2 and 10. To immortalize MEFs, p53 was inactivated by CRISPR-Cas9 mutagenesis using p53-targeting sgRNA, sgp53, listed in Table S1, expressed from BRD005 (U6-sgRNA-spCas9-RFP) (a gift from the Broad Institute, MIT). Briefly, MEFs were transiently transfected with sgp53-expressing BRD005 using the Fast-forward Attractene (301005; Qiagen) protocol and passaged to select for immortalized clones. Immortalized MEFs were maintained in 10% fetal bovine serum, 50 U ml^−1^ penicillin-streptomycin, 2 mM L-glutamine, and 0.1 mM nonessential aa. hTERT RPE-1 cells, a gift from Dr. David Breslow (Yale University, New Haven, CT, USA), were maintained in DMEM:F12 (Gibco), supplemented with 10% fetal bovine serum, 50 U ml^−1^ penicillin-streptomycin, and 2 mM L-glutamine. PC9 cells, a gift from Dr. Katerina Politi (Yale University, New Haven, CT, USA), were maintained in RPMI (Gibco) supplemented with 10% fetal bovine serum and 50 U ml^−1^ penicillin-streptomycin.

*Malat1* PiggyBac expression vectors were prepared by cloning commercially synthesized *Malat1* sequences (IDT) downstream of the Doxy-inducible promotor in the PB-TRE-EGFP-EF1a-rtTA vector (Addgene, 104454) after excision of the EGFP sequence. FL *Malat1* contained sequences A–E, while ΔA, ΔB, ΔC, and ΔD deletion mutant constructs lacked regions A through D, respectively, and Δ9xATG contained 9 ATG->AAG mutations (NR_002847.3: T210A, T271A, T567A, T742A, T1512A, T1718A, T1901A, T2206A, and T3144A). The 9 sORFs were selected based on length exceeding 20 aa, presence of strong Kozak sequences, and location in the 5′ half of *Malat1*, which has a strong ribosomal footprint. All constructs were verified by restriction digest and Sanger sequencing. *Malat1* FL, mutant, and deletion constructs were co-transfected with PiggyBac transposase vector into *Malat1* KO MEFs using Attractene transfection reagent (301005; Qiagen) according to the Fast-Forward Protocol described by the manufacturer. Selection in 2 µg/ml puromycin (P9620; Millipore-Sigma) was initiated at 48 h after transfection and continued for 3–4 days. Exogenous *Malat1* expression was initiated through treatment with 1 µg/ml doxycycline (D1822; Millipore-Sigma).

FUCCI mAG-hGem(1/110) (Sakaue-Sawano et al., 2008) was cloned into MSCV-puro (68469; Addgene) and transfected via calcium phosphate into the Phoenix-ECO retrovirus producer cell line (CRL-3214; ATCC). Cells were infected by three rounds of retroviral infection performed 8–16 h apart by passing viral-containing supernatant through a 0.45-µM filter (28145-505; VWR), supplemented with 4 µg/ml polybrene (107689; Millipore-Sigma). Cells were selected in 2 µg/ml puromycin (P9620; Millipore-Sigma).

### Drug treatments

Cells were incubated for the indicated timepoints with the following drugs: 50 ng/ml Nocodazole (AC358240100; Thermo Fisher Scientific), 8 μM RO-3306 (15149; Cayman Chemical Company), 1 μg/ml Doxycycline (D1822; Millipore-Sigma), 0.5 μM Doxorubicin (D1515; Millipore-Sigma), 5 μg/ml Actinomycin D (C988H61; Millipore-Sigma), 50 μg/ml Cycloheximide (C974S42; Millipore-Sigma), 5 μM hSmg1 inhibitor 11e (HY-124760; MedChemExpress), and 100 nM Osimertinib (AZD9291, 16237; Cayman Chemical Company).

### Cell cycle synchronization

Mitosis-enriched cell populations were generated by treatment with 50 ng/ml Nocodazole (AC358240100; Thermo Fisher Scientific) for 4 h, followed by manual shake-off of mitotic cells in PBS. To initiate recovery from mitotic arrest, cells were washed once in PBS and replated in normal growth medium for the indicated release time. At indicated time points, adherent and non-adherent cells were harvested for RNA and protein analyses or fixed for smRNA-FISH.

### smRNA-FISH

smRNA-FISH was performed according to the manufacturer recommendations using commercially available probes, specific to murine *Malat1* (Q570, SMF-3008-1, and Q670; Biosearch Technologies, custom made, same probes as SMF-3008-1), murine Neat1-5′ (Q570 SMF-3009-1; Biosearch Technologies), and human *MALAT1* (Q570, SMF-2035-1; Biosearch Technologies). Briefly, cells were grown on coverslips and fixed for 10 min in 4% methanol-free formaldehyde (Thermo Fisher Scientific) diluted in RNase-free 1xPBS (Thermo Fisher Scientific) at RT, followed by 1xPBS washes. Cells were dehydrated overnight at 4°C in 70% EtOH diluted in DEPC-H_2_O and stored in 70% EtOH for up to a week at 4°C. Coverslips were transferred to a hybridization chamber and equilibrated for 5 min in Wash Buffer A (SMF-WA1-60; Biosearch Technologies) prepared with formamide (Millipore Sigma) according to the manufacturer’s instructions. Cells were incubated overnight at 30°C with the indicated probes diluted 1:50 in Hybridization solution (SMF-HB1-10; Biosearch Technologies). The next day, cells were washed two times for 30 min at 30°C in Wash Buffer A, incubated in Wash Buffer B (SMF-WB1-20; Biosearch Technologies) for 5 min at RT, mounted in antifade reagent (Vectashield Mounting medium with DAPI, Vector Laboratories), and sealed with nail polish.

### Combined IF-smRNA-FISH

Cells, grown on coverslips, were fixed in 4% methanol-free formaldehyde (Thermo Fisher Scientific) diluted in RNase-free 1xPBS (Thermo Fisher Scientific) for 10 min at RT, washed twice with 1xPBS, permeabilized with 0.5% Triton X-100 diluted in 1xPBS for 10 min at RT, and washed three times with 1xPBS. Next, cells were blocked for 10 min in 4% BSA (Jackson ImmunoResearch) in 1xPBS prior to incubation with anti-Srrm2 primary antibody (1:500, NBP2-55691; Novus) diluted in 1xPBS for 2 h at RT. Following three 1xPBS washes, cells were incubated with Alexa Fluor 488 AffiniPure Goat Anti-Rabbit IgG (H+L) secondary antibody (1:500, Jackson ImmunoResearch) diluted in 1xPBS for 1 h at RT, followed by two 1xPBS washes. Cells were fixed in 4% formaldehyde for 10 min at RT, followed by two 1xPBS washes, prior to performing smRNA-FISH, as described above.

### Image acquisition and analysis

smRNA-FISH and IF-smRNA-FISH imaging was performed on an Axio Imager 2 microscope system (Zeiss) with a Plan-Apochromat 63×1.4 oil DIC objective lens (Zeiss) and a Plan-Apochromat 40× 1.3 oil DIC (UV) VIS-IR objective lens (Zeiss) using Immersol 518F Immersion Oil (Zeiss). Images were captured with an Axiocam 503 mono camera using Zen 2.6 Pro software (Zeiss) with the following settings: (Binning—2 × 2; filter [target]: exposure—Cy5 [*Malat1* Q670]: 1,000 ms; rhodamine [*Malat1*, *MALAT1*, and *Neat1-5*′ Q570]: 1,000 ms; EGFP (Srrm2): 200 ms; DAPI [DNA]: 20 ms). Quantification of *Malat1/MALAT1* nuclear mean intensity (MI) was performed in Zen software using the measurement tool within a circle with 14.6 μm diameter in a.u. MI of an equivalent area was subtracted as a background in each field. Cells were scored as positive for cytoplasmic *Malat1* if they had 50 or more *Malat1* cytoplasmic foci, representative of single molecules. This quantification was appropriate as the presence or absence of cytoplasmic *Malat1* molecules was largely binary. 50 was selected as the cutoff to account for the number of specific foci commonly captured in focus in a single plane. Images were exported as TIFF files and edited in Adobe Photoshop.

### RNA isolation and qRT-PCR

Total RNA was isolated with the RNeasy Mini Kit (Qiagen). 1 μg of total RNA was reverse transcribed using the High Capacity cDNA Reverse Transcription Kit (Applied Biosystems). For ActD experiments, on-column digestion with DNase was performed using the RNase-Free DNase Set (Qiagen, 79254). SYBR Green PCR master mix (Applied Biosystems) was used for quantitative PCR in triplicate reactions with primers listed in Table S1. Relative RNA expression levels were calculated using the ddCt method relative to *Gapdh* or *Atp5f1e* (for ActD experiments) and normalized to control samples, when indicated.

### Immunoblotting

Cells were collected, counted, and lysed in 2 × Laemmli buffer (100 mM Tris-HCl pH 6.8, 200 mM DTT, 3% SDS, 20% (wt/vol) glycerol, and 0.01% bromophenol blue) at 1 × 10^4^ cells/μl. Samples were heated at 95°C for 7 min and passed through an insulin syringe. Protein from 1 × 10^5^ cells was separated on a 10% SDS-polyacrylamide gel and transferred to a nitrocellulose membrane (Bio-Rad). After blocking (5% milk, PBST), membranes were incubated overnight at 4°C in primary antibodies anti-cyclin B1 (1:1,000, 4138S; Cell Signaling Technology) and anti-Hsp90 (1:1,000, 610419; BD Biosciences), then 1 h at RT in secondary antibody (1:10,000, Peroxidase AffiniPure Donkey Anti-Rabbit IgG [H+L] and Peroxidase AffiniPure Goat Anti-Mouse IgG [H+L], Jackson ImmunoResearch). Protein bands were visualized using Pierce ECL Prime Western Blotting Substrate (Thermo Fisher Scientific).

### Cell cycle analysis by FACS

Trypsinized cells were pelleted, washed in PBS once, fixed by dropwise resuspension in ice-cold 70% ethanol, and incubated overnight at 4°C. Fixed cells were stained in PBS with 0.5% BSA (A7638; Millipore Sigma), 25 µg/ml propidium iodide (P3566; Thermo Fisher Scientific), and 100 µg/ml RNase A (19101; Qiagen) at RT for 4 h. Propidium iodide signal was detected in the PE Texas Red channel with an LSRFortessa X-20 flow cytometer (BD Biosciences).

### Subcellular fractionation

Cytoplasmic and nuclear RNA were isolated by subcellular fractionation. Briefly, cell pellets were resuspended in 175 μl of ice-cold Buffer RLN (50 mM Tris-HCl, pH 8.0, 140 mM NaCl, 1.5 mM MgCl_2_, and 0.5% [vol/vol] Nonidet P-40) containing 1,000 U/ml RNase inhibitor (M0314S; NEB) and 1 mM DTT and incubated on ice for 5 min to lyse the plasma membrane. The lysate was centrifuged, and the supernatant containing the cytoplasmic fraction was transferred to a new tube for cytoplasmic RNA isolation. The remaining pellet containing intact nuclei was washed with ice-cold Buffer RLN to remove residual cytoplasmic contamination and processed for nuclear RNA isolation using the RNeasy Mini Kit (Qiagen). Relative RNA expression levels were calculated using the ddCt method for *Malat1*, *Rn7sl* (cytoplasmic marker), or *Kcnq1ot1* (nuclear marker) relative to *Gapdh*.

### RNA stability assay

RNA stability assay was performed using the Click-iT Nascent RNA Capture Kit (C10365; Invitrogen) according to the manufacturer’s protocol. 200,000 MEFs were plated in 35-mm dishes. The following day, cells were incubated for 1 h with 0.5 mM 5-EU, then media containing EU was removed, and cells were rinsed and then grown in fresh media until harvest at 0, 4, 8, and 24 h following EU removal. Total RNA was extracted using TRIzol and nascent RNA capture was performed according to the manufacturer’s recommendations using the following specifications: EU biotinylation was performed using 1 μg total RNA and 0.5 mM biotin azide. Biotinylated RNA was precipitated overnight at −80°C and 0.5 μg RNA was captured using 50 μl of Dynabeads MyOne Streptavidin T1 magnetic beads. RT-PCR was performed with the SuperScript VILO cDNA synthesis kit (11756050; Invitrogen) using bead-bound RNA, and qRT-PCR was done using the SsoAdvanced Universal SYBR Green Supermix (1725274; Bio-Rad) according to the manufacturer’s instructions.

### Quantification and statistical analysis

Data are represented as mean ± SD of the indicated number of replicates. Paired or unpaired *t* test, as indicated, was performed to test for statistical significance between pairs of samples and indicated as follows: *ns*, not significant; *P < 0.05; **P < 0.01; ***P < 0.001.

### Online supplemental material

Fig. S1 describes copy number quantification of *Malat1* abundance and demonstrates similar cell cycle–dependent *Malat1/MALAT1* localization patterns in murine and human cell lines, related to Figs. 1 and 2. Fig. S2 provides supporting information for translation inhibition studies, described in Fig. 3, as well as demonstrates that Neat1_2/NEAT1_2 is not subject to the cell cycle– and translation-mediated regulatory mechanism that controls *Malat1/MALAT1* abundance. Fig. S3 shows characterization of the localization and expression levels of the *Malat1* WT and mutant complementation constructs used in Fig. 4. Table S1 shows primers and sgRNA used in this study.

## Declaration of generative AI and AI-assisted technologies

Authors declare that generative AI and AI-assisted technologies were not used in this manuscript.

## Supplementary Material

Review History

Table S1 shows primers and sgRNA used in this study.

SourceData F2 is the source file for Fig. 2.

## Data Availability

The data are available from the corresponding author upon request.
