# Supplementary material for: Cell cycle–dependent translation-mediated turnover of the long noncoding RNA Malat1
Source: J Cell Biol. 2026 Aug 25;225(10):e202512059. doi: 10.1083/jcb.202512059 (PMC13505482; doi:10.1083/jcb.202512059)
Supplement: Supplementary file 1 [file jcb_202512059_review_history.pdf]

JCB

Cell cycle-dependent translation-mediated turnover of the long noncoding RNA Malat1

Leah Plasek-Hegde, Yeolhoe Kim, Rahul Gupta, Emily Dangelmaier, Sigrid Nachtergaele, and Nadya Dimitrova

Corresponding Author(s): Nadya Dimitrova, Yale University

Review Timeline:

Submission Date:	2025-12-12
Editorial Decision:	2026-01-13
Revision Received:	2026-06-25
Editorial Decision:	2026-07-13
Revision Received:	2026-08-04

Monitoring Editor: Jens Lykke-Andersen

Scientific Editor: Gabriele Stephan

Transaction Report:

(Note: With the exception of the correction of typographical or spelling errors that could be a source of ambiguity, letters and reports are not edited. The original formatting of letters and referee reports may not be reflected in this compilation.)

DOI: https://doi.org/10.1083/jcb.202512059

January 13, 2026

Re: JCB manuscript #202512059

Nadya Dimitrova
Yale University

Dear Dr. Dimitrova,

Thank you for submitting your manuscript entitled "Cell cycle-dependent translation-mediated turnover of the long noncoding RNA Malat1". The manuscript was assessed by expert reviewers, whose comments are appended to this letter. We invite you to submit a revision if you can address the reviewers' key concerns, as outlined here.

You will see that the reviewers are overall supportive and provide constructive feedback. We consider it particularly important to address the comments that focus on strengthening the evidence that the degradation mechanism is indeed NMD and that this is a general phenomenon of MALAT1 regulation beyond MEFs, while detailing the specific mechanism of exactly how NMD is triggered is less important given that this is submitted as a Report.

While you are revising your manuscript, please also attend to the following editorial points to help expedite the publication of your manuscript. Please direct any editorial questions to the journal office.

GENERAL GUIDELINES:
Text limits: Character count for a Report is < 20,000, not including spaces. Count includes title page, abstract, introduction, the joint Results & Discussion, and acknowledgments. Count does not include materials and methods, figure legends, references, tables, or supplemental legends.

Figures: Reports may have up to 5 main text figures. To avoid delays in production, figures must be prepared according to the policies outlined in our Instructions to Authors, under Data Presentation, https://jcb.rupress.org/site/misc/ifora.xhtml. All figures in accepted manuscripts will be screened prior to publication.

IMPORTANT: It is JCB policy that if requested, original data images must be made available. Failure to provide original images upon request will result in unavoidable delays in publication. Please ensure that you have access to all original microscopy and blot data images before submitting your revision.

Supplemental information: There are strict limits on the allowable amount of supplemental data. Reports may have up to 3 supplemental figures. Up to 10 supplemental videos or flash animations are allowed. A summary of all supplemental material should appear at the end of the Materials and methods section.

Please note that JCB now requires authors to submit Source Data used to generate figures containing gels and Western blots with all revised manuscripts. This Source Data consists of fully uncropped and unprocessed images for each gel/blot displayed in the main and supplemental figures. For assays performed using capillary electrophoresis and/or immunoassay-based detection, authors should instead provide the electropherogram graph(s) for each experiment, plotting fluorescence/chemiluminescence intensity vs. molecular weight/size. Please be sure to provide one Source Data file for each figure gels, blots, and/or capillary electrophoresis assays along with your revised manuscript files. File names for Source Data figures should be alphanumeric without any spaces or special characters (i.e., SourceDataF#, where F# refers to the associated main figure number or SourceDataFS# for those associated with Supplementary figures). For traditional gels and blots, the lanes of the gels/blots should be labeled as they are in the associated figure, the place where cropping was applied should be marked (with a box), and molecular weight/size standards should be labeled wherever possible. For capillary electrophoresis assays, each trace in the graph should be color-coded and labeled to indicate which protein, gene, or sample is being measured (please try to avoid red/green combinations to accommodate our color-blind readers).

Source Data files will be made available to reviewers during evaluation of revised manuscripts and, if your paper is eventually published in JCB, the files will be directly linked to specific figures in the published article.

Source Data Figures should be provided as individual PDF files (one file per figure). Authors should endeavor to retain a minimum resolution of 300 dpi or pixels per inch. Please review our instructions for export from Photoshop, Illustrator, and PowerPoint here: https://rupress.org/jcb/pages/submission-guidelines#revised

The typical timeframe for revisions is three to four months. If you anticipate any difficulties in meeting this aforementioned revision time limit, please contact us and we can work with you to find an appropriate time frame for resubmission. Please note that papers are generally considered through only one revision cycle, so any revised manuscript will likely be either accepted or rejected.

When submitting the revision, please include a cover letter addressing the reviewers' comments point by point. Please also highlight all changes in the text of the manuscript.

We hope that the comments below will prove constructive as your work progresses. We would be happy to discuss them further once you've had a chance to consider the points raised in this letter.

Thank you for this interesting contribution to Journal of Cell Biology. You can contact us at the journal office with any questions at cellbio@rockefeller.edu.

Sincerely,

Jens Lykke-Andersen
Monitoring Editor
Journal of Cell Biology

Gabriele Stephan
Scientific Editor
Journal of Cell Biology

Reviewer #1:

The authors address how expression of the long noncoding RNA Malat1 is controlled. It has long been known that Malat1 is highly expressed in normal cells and that it becomes even more abundant in cancer cells. The current work now surprisingly suggests that Malat1 is degraded in the cytoplasm in early G1, thereby resetting Malat1 levels after each cell cycle. Such degradation requires NMD and translation, suggesting a role for sORF translation although this part of the model has not been explicitly tested. I find the overall ideas in this manuscript to be very interesting but the authors should further prove the generality of this mechanism in other cell lines and dive further into the proposed NMD mechanism.

(1) Fig 1: All the data shown are from MEFs. The authors' conclusions would be significantly strengthened if they could demonstrate a clear connection between cell cycle stage and Malat1 levels in a panel of other cell lines, including human.
(2) Fig 3C: It would be helpful to include images with more cells in them so that a reader can better judge the percentage of cells with cytoplasmic Malat1. The authors show only 2 cells in most of the current images.
(3) Fig 4: The NMD inhibition results are very interesting but the authors have not tried to remove any of the sORFs, e.g. the M1 ORF, to determine if that is sufficient to increase Malat1 levels. Such experiments would directly test the author's underlying model for how Malat1 levels are controlled.

Minor points:
(1) Introduction: The authors write that RNase P processing is a post-transcriptional step that cleaves polyadenylated nascent Malat1. The authors can correct me if I am wrong but I am not familiar with any evidence of such a stepwise model of polyadenylation followed by RNase P processing.
(2) Introduction: Screening by Che et al 2025 Nature Communications 16:4741 has recently revealed some factors that appear to control Malat1 degradation. This work should be mentioned.
(3) Figure 1D: Please clarify how high vs low Malat1 nuclear intensity was determined. It should likewise be clarified how cells with cytoplasmic Malat1 was calculated in Figs 3 and 4.
(4) Fig 2A: Some of the white arrows appear to be misplaced.
(5) Fig 3A: Transcript should be labeled starting at 1, not 0.

Reviewer #2:

The manuscript addresses the cell cycle-dependent degradation mechanism of cytoplasmic MALAT1 lncRNA. During mitosis, when nuclear structures transiently disassemble, nuclear-localized RNAs exhibit diverse behaviors. The highly abundant MALAT1 lncRNA, which is normally localized to nuclear speckles, becomes dispersed into the cytoplasm during mitosis. However, the fate of cytoplasmic MALAT1 after completion of cell division has not been well characterized. This study reveals that cytoplasmic MALAT1 is rapidly degraded during early G1 phase via a translation-dependent RNA decay pathway, presumably nonsense-mediated decay (NMD). This finding provides a consistent and straightforward explanation for the fate of cytoplasmic MALAT1 during the cell cycle. While the study is potentially interesting, several issues related to experimental interpretation, quantification criteria, and statistical analysis need to be addressed before the conclusions can be fully supported. With appropriate revisions, the manuscript could be suitable for acceptance as a Report in The Journal of Cell Biology.

Major comments

1. Figure S1: It is unclear whether the 3′ read-through signals can be directly interpreted as a proxy for nascent transcription or transcription rate. This assumption should be clarified and justified.
2. Figures 1, 2D, and 4D: Changes in "nascent" MALAT1 levels are observed under various treatments (e.g., RO-3306, nocodazole, cycloheximide), but their interpretation is not sufficiently discussed. The authors should clarify why nascent MALAT1 signals increase or decrease under these conditions and whether these treatments affect MALAT1 transcription, RNA processing, or both.
3. Figure 2: The claim that the two bright nuclear dots represent nascent MALAT1 transcription sites requires experimental support. Confirmation by DNA-FISH or RNA-FISH using probes targeting the 3′ downstream region of MALAT1 (or even the adjacent NEAT1 locus) would strengthen this interpretation.
4. Figures 3 and 4: Treatments such as ΔA expression, NMD inhibition, and CHX + nocodazole increase cytoplasmic and/or total MALAT1 levels. The authors should exclude the possibility that these effects are secondary to alterations in cell cycle distribution. Assessing cell cycle profiles under each condition using the FUCCI system or established cell cycle markers would strengthen the conclusions.
5. Figures 4B and 4C: Nuclear MALAT1 levels should be assessed to determine whether cytoplasmic MALAT1 can be re-imported into the nucleus, or whether nuclear MALAT1 levels are predominantly determined by newly synthesized transcripts.

Minor comments

1. The Introduction mentions miRNA-mediated regulation as a known cytoplasmic degradation pathway for MALAT1, whereas this study identifies involvement of NMD. The relationship between these pathways should be discussed-whether they are mutually exclusive, act independently, or are context-dependent.
2. In Figure 1D, cells are classified as "Low" or "High" MALAT1 based on nuclear signal intensity, but the threshold used for this classification is not defined. The criteria should be explicitly stated in the Methods or figure legend. Ideally, a histogram of signal intensity distribution should be provided as a supplemental figure to justify the classification.
3. In Figure S1D, the reported difference between early- and late-passage MEFs is difficult to assess from the current images. Wider field-of-view images showing multiple cells simultaneously would better support this claim.
4. In Figures 3D, 4B, and 4C, the percentage of cells positive for cytoplasmic MALAT1 is quantified, but the criteria used to define "cytoplasmic MALAT1-positive" cells are not described. The quantification protocol (e.g., signal intensity threshold, localization pattern, nuclear-to-cytoplasmic ratio, background correction) should be clearly stated in the Methods or figure legends. Because this categorical classification is highly dependent on threshold definition and does not necessarily reflect quantitative RNA changes, complementary quantitative analyses should be included, such as qPCR of cytoplasmic fractions and/or quantification of cytoplasmic MALAT1 signal intensity. In addition, the number of cells analyzed per experiment should be reported to assess the robustness of the data.
5. Cytoplasmic MALAT1 has been reported to interact with Staufen, suggesting a possible contribution of Staufen-mediated decay (SMD). The Discussion should therefore address the potential involvement of SMD, in addition to NMD, in cytoplasmic MALAT1 regulation.
6. Statistical analysis
Multiple comparisons are analyzed using t-tests, which is not appropriate. Proper multiple-comparison corrections or alternative statistical methods should be applied. Data variability should be presented as standard deviation (SD) rather than standard error of the mean (SEM), unless a clear rationale for SEM is provided. In several figures (e.g., Figures 2D, 3B, and 4B), statistical significance is not consistently indicated. While not mandatory, including statistical annotations where applicable would improve clarity and consistency.
7. Several abbreviations (e.g., ENE, FUCCI system) are introduced without definition and should be explained at first mention.

Reviewer #3:

In this manuscript, Plasek-Hegde and colleagues investigate mechanisms that regulate Malat1 abundance in cells with different proliferation rates. Using MEFs, they find that cells that proliferate slowly accumulate higher levels of Malat1 than rapidly dividing cells. Cell cycle inhibition and RNA-FISH implicate degradation of cytoplasmic Malat1 upon exit from M phase as a major mechanism responsible for reducing its cellular abundance. Of a series of Malat1 deletion mutants, one lacking a previously identified 5' ORF accumulates to the highest levels and shows increased cytoplasmic localization, suggesting a translation-dependent decay mechanism. To test this, they inhibit NMD using a small molecule NMDi-14 and translation with cycloheximide, both of which rescue RNA abundance in asynchronous cells and in G1. Finally, they show that PC9 cells induced to enter a slow-cycling state express higher levels of MALAT1. This is an interesting manuscript that provides an unexpected mechanism for regulation of a predominantly nuclear "non-coding" RNA. While the available data are highly suggestive, more work is needed to provide fully convincing evidence. In addition, the presentation could be improved in several places by clearer reporting (particularly improving the very sparse figure legends) and improved quantitative methods. Please see below for specific comments:

1. The authors show that NMD inhibition (Figure 4) and deletion of the 5' end (deltaA; Figure 3) both increase Malat1 abundance, but they do not demonstrate that A) deletion of the 5' end abolishes NMD sensitivity or that B) the wild-type

construct and the remaining deletions are NMD-sensitive. In addition, mutants that specifically remove putative ORF M1 should be tested to fully substantiate the authors' claims.
2. The only evidence provided for NMD involvement is NMDi-14 treatment. One or more additional methods should be provided (e.g. SMG1 inhibitor and/or NMD factor knockdowns) to supplement the use of what remains a fairly poorly characterized drug. In addition, it is important to show that decay is actually occurring under normal conditions--this should be done with 4SU or other metabolic labeling-based approaches (such as Roadblock-PCR), not with Actinomycin D treatment, which causes profound cellular stress.
3. A previous study (PMID: 33784500) showed that a truncated, polyadenylated isoform of Malat1 can be produced under certain conditions. The qPCR approach taken by the authors cannot distinguish among different potential Malat1 isoforms that might be produced upon cellular perturbations or upon mutation of reporter constructs. Northern blotting or sequencing-based methods capable of detecting the full array of Malat1 isoforms expressed from the endogenous locus and from the reporter constructs are needed. Also related to this point, I was not able to discern the PCR primers used for the quantification of RNA levels in Figure 3B. The primers listed in the supplementary table both recognize regions of Malat1 that are absent in at least one deletion construct. Please clarify and ensure that the methods used are capable of detecting all mutants equally.
4. Also in Figure 3B, the experiments are performed in asynchronous cells, but the magnitude of the effect of the deltaA deletion greatly exceeds the effects observed in any of the experiments involving the endogenous Malat1. Can the authors comment on why this might be the case?
5. I'm surprised by the small decreases observed in "nascent" Malat1 levels in M phase (Figure 1E, Figure 2D, 4D). There should not be ongoing transcription in M phase, so it seems that the RNA detected may be unprocessed rather than nascent or that there may be a problem with the qPCR assay used (it can be difficult to accurately quantify low-abundance downstream products). In addition, it is quite unexpected that the levels of "nascent" RNA would significantly increase with cycloheximide treatment (Figure 4D). Can the authors explain this observation?
6. In several places, quantification and methods reporting are lacking:
a. Figure 1D: What defines "high" and "low" signal? It would be much better to show distributions of signal intensities rather than taking this binning approach, which seems to exaggerate the magnitude of the differences across the cell cycle.
b. Figure S1D: Quantification is needed to substantiate the claim in the text that early-passage cells show more variability in nuclear RNA abundance than late-passage cells.
c. Figure 4B and C: How was the cutoff drawn to determine cells with positive/negative cytoplasmic signal? Again, a distribution of intensities would be preferable here.
d. Throughout, figure legends require reporting of exact replicate numbers, numbers of cells counted per replicate, and methods used to determine error bars and p-values. SD rather than SEM should be used.
e. Figure 3C, the authors state that they "observed [sic] high rate of co-occurrence of abundant nuclear and cytoplasmic Malat1 staining...," but no quantification is provided to substantiate this claim.

Minor points:

1. The idea that NMD takes place in the pioneer round of translation is controversial and should not be stated as fact (see for example: PMIDs: 23665581, 23665580, 31155380).
2. Please ensure original discovery papers are cited alongside recent reviews in all sections (e.g., FUCCI, smFISH/IF methods, NMD models) and add missing primary references where appropriate.
3. The authors write that there "were no consistent changes in nascent Malat1 (Figure 1E)," but the figure shows significant differences among cycling, M-phase, and G2 phase conditions.
4. Figure 2A shows FISH of fixed cells, so it's not clear to me how the authors determine that the Malat1 signal "abruptly disappeared."

Response to Reviewers

We are grateful to the Reviewers for their thoughtful and insightful comments on the manuscript. To address the comments and concerns, we performed multiple additional experiments as well as improved quantification. We present new data and analyses in revised figures that significantly strengthen the conclusions for cell cycle-dependent and translation-mediated degradation of *Malat1*. We have also extensively updated the text in response to Reviewers' feedback and recommendations. Below, we have provided a point-by-point responses to each Reviewer's comments.

Reviewer #1:

The authors address how expression of the long noncoding RNA Malat1 is controlled. It has long been known that Malat1 is highly expressed in normal cells and that it becomes even more abundant in cancer cells. The current work now surprisingly suggests that Malat1 is degraded in the cytoplasm in early G1, thereby resetting Malat1 levels after each cell cycle. Such degradation requires NMD and translation, suggesting a role for sORF translation although this part of the model has not been explicitly tested. I find the overall ideas in this manuscript to be very interesting but the authors should further prove the generality of this mechanism in other cell lines and dive further into the proposed NMD mechanism.

We thank the Reviewer for highlighting the significance of our finding. We believe that the revised manuscript strengthens the conclusion that cell cycle- and translation-dependent regulation of *Malat1* is a general mechanism that occurs across different cell lines and species.

(1) Fig 1: All the data shown are from MEFs. The authors' conclusions would be significantly strengthened if they could demonstrate a clear connection between cell cycle stage and Malat1 levels in a panel of other cell lines, including human.

We agree with the Reviewer that it is important to determine whether or not our conclusions are limited to MEFs. To this end, we extended all key findings to the human cell line RPE as well as presented data in the patient-derived lung adenocarcinoma cell line PC9. We report increased *MALAT1* levels in RPE cells arrested in G2/M or M phases relative to asynchronous cells, consistent with the cell cycle-dependent regulation of *Malat1* in MEFs (**Fig. 1J**). Additionally, we performed smRNA-FISH to visualize the heterogeneity of *MALAT1* levels in asynchronous RPE and PC9 cell populations, and we confirmed localization of *MALAT1* in the cytoplasm of cells that cytologically appear to be in early G1, as observed in MEFs (**Fig. S1E-I**). Lastly, we performed cell cycle synchronization, mitotic shake-off and release experiments in RPE cells. Similarly to MEFs, we observed that *MALAT1*, but not *NEAT1_2*, levels significantly decreased at 2 hours post mitosis and that this decrease is rescued by inhibition of translation (**Fig. 3C, S2D)**. Together, these data support shared mechanisms of cell cycle- and translation-dependent regulation of murine and human *Malat1/MALAT1.*

(2) Fig 3C: It would be helpful to include images with more cells in them so that a reader can better judge the percentage of cells with cytoplasmic Malat1. The authors show only 2 cells in most of the current images.

We thank the Reviewer for suggesting inclusion of panoramic images to illustrate our findings. We have included such images in several figures (**Fig. 1D; S1E, G; 4B, F**). These images illustrate the extent of *Malat1/MALAT1* signal heterogeneity as well as provide representative examples of the frequency of cells with cytoplasmic signal.

(3) Fig 4: The NMD inhibition results are very interesting but the authors have not tried to remove any of the

sORFs, e.g. the M1 ORF, to determine if that is sufficient to increase Malat1 levels. Such experiments would directly test the author's underlying model for how Malat1 levels are controlled.

We agree with the Reviewer that the ∆A mutant encompasses a very large region, which in addition to the M1 small ORF (sORF), may harbor additional functional elements. To address the contribution of sORFs to *Malat1* degradation, we sought to develop a mutant of *Malat1* with impaired sORFs. This was very challenging due to the presence of a large number of sORFs in *Malat1* (41 sORFs longer than 20 aa, 55 sORF longer than 15 aa). We focused on sORFs in the 5' half of *Malat1*, where most of the ribosomal footprints have been reported, and we selected to mutate the ATGs of 9 sORFs that harbored strong Kozak sequences, including M1. In the revised manuscript, we report that the resulting ∆9xATG *Malat1* mutant is significantly more abundant compared to FL *Malat1*, consistent with a contribution of sORFs to the regulation of *Malat1* abundance (**Fig. S3E).**

While region A and the 9 sORFs were found to contribute to *Malat1* degradation, we also observed that they were not required. Specifically, we report that while degradation of ∆A *Malat1* transcripts in the early G1 cytoplasm was reduced compared to FL *Malat1* (**Fig. 4A, E)**, ∆A *Malat1* transcripts still underwent significant degradation through a translation-dependent mechanism (**Fig. 4A, E-G**). Our interpretation of these data is that *Malat1* harbors multiple redundant degradation elements. We believe that downstream (B-D) regions of *Malat1* also harbor elements that contribute to *Malat1* decay.

Minor points:
(1) Introduction: The authors write that RNase P processing is a post-transcriptional step that cleaves polyadenylated nascent Malat1. The authors can correct me if I am wrong but I am not familiar with any evidence of such a stepwise model of polyadenylation followed by RNase P processing.

We thank the Reviewer for correcting our misconception. There is indeed no evidence for the production of polyadenylated *Malat1*, which is then processed by RNAseP. We have rephrased our introduction.

(2) Introduction: Screening by Che et al 2025 Nature Communications 16:4741 has recently revealed some factors that appear to control Malat1 degradation. This work should be mentioned.

We now cite this work. However, we also note that the *Malat1* construct employed in this study lacks ENE, and therefore, the degradation pathways identified in this study act on unprotected *Malat1*. The relevance of these findings to the regulation of endogenous *Malat1* is not clear.

(3) Figure 1D: Please clarify how high vs low Malat1 nuclear intensity was determined. It should likewise be clarified how cells with cytoplasmic Malat1 was calculated in Figs 3 and 4.

We thank all three Reviewers for highlighting this omission in our analysis. To strengthen our descriptive conclusions, we have now performed detailed quantitative analysis.

We measured **nuclear *Malat1* signal** using the Mean Intensity tool in Zen on images acquired with consistent exposure and corrected for background, as described in the revised Methods. This analysis allowed us to compare the distribution and strength of nuclear *Malat1* signal intensity in early vs late passage MEFs (**Fig. 1E**), in early G1 vs G1/S/G2 MEFs (**Fig. 1F, H**) and RPE cells (**Fig. S1F**), and in knockout MEFs rescued with FL vs mutant *Malat1* (**Fig. 4C, G**).

We quantified **the fraction of cells with cytoplasmic *Malat1*** by counting cells as positive if they had 50 or more discrete single molecule *Malat1* foci in the cytoplasm. This categorical classification was appropriate as

the presence or absence of *Malat1* in the cytoplasm of WT MEFs or RPE cells was a largely binary occurrence with cells either lacking cytoplasmic foci or harboring hundreds of discrete cytoplasmic foci. The cells with cytoplasmic *Malat1* signal were consistently cytologically identifiable as being in the early G1 stage of the cell cycle, based on the proximity of daughter nuclei. We selected 50 cytoplasmic foci as the threshold to account for the number of foci commonly captured in focus in a single-plane image. This quantification has been included in several figures (**Fig. 1F, S1F, 3E, 4F**).

(4) Fig 2A: Some of the white arrows appear to be misplaced.

Fixed. Thank you!

(5) Fig 3A: Transcript should be labeled starting at 1, not 0.

Numbering has been updated. Thank you!

Reviewer #2:

The manuscript addresses the cell cycle-dependent degradation mechanism of cytoplasmic MALAT1 lncRNA. During mitosis, when nuclear structures transiently disassemble, nuclear-localized RNAs exhibit diverse behaviors. The highly abundant MALAT1 lncRNA, which is normally localized to nuclear speckles, becomes dispersed into the cytoplasm during mitosis. However, the fate of cytoplasmic MALAT1 after completion of cell division has not been well characterized. This study reveals that cytoplasmic MALAT1 is rapidly degraded during early G1 phase via a translation-dependent RNA decay pathway, presumably nonsense-mediated decay (NMD). This finding provides a consistent and straightforward explanation for the fate of cytoplasmic MALAT1 during the cell cycle. While the study is potentially interesting, several issues related to experimental interpretation, quantification criteria, and statistical analysis need to be addressed before the conclusions can be fully supported. With appropriate revisions, the manuscript could be suitable for acceptance as a Report in The Journal of Cell Biology.

We thank the Reviewer for their careful consideration of our manuscript and for the helpful suggestions how to improve the rigor of our work.

Major comments

1. Figure S1: It is unclear whether the 3′ read-through signals can be directly interpreted as a proxy for nascent transcription or transcription rate. This assumption should be clarified and justified.

We agree with the Reviewer that we made an assumption that the signal from 3' primers can be used as a proxy for nascent transcription. 3' products could reflect a combination of transcription and processing rates. We have renamed the primers as primer sets A through E to reflect their positions within the *Malat1* transcript and clarified that primers, specific to region E, detect unprocessed transcripts (**Fig. 1A**).

2. Figures 1, 2D, and 4D: Changes in "nascent" MALAT1 levels are observed under various treatments (e.g., RO-3306, nocodazole, cycloheximide), but their interpretation is not sufficiently discussed. The authors should clarify why nascent MALAT1 signals increase or decrease under these conditions and whether these treatments affect MALAT1 transcription, RNA processing, or both.

This is a very important point, and we are grateful to the Reviewer for bringing it up. We find that transcripts detected with primer set E are several hundred-fold less abundant compared to transcripts detected with primer sets A-D, indicating efficient *Malat1* processing. To reflect this difference, we plotted relative rather than

normalized levels and show that mature *Malat1* transcripts are the predominant species **(Fig. 1B, C and Fig. 2F**). We believe that the inconsistent fold change differences, we previously reported when plotting normalized *Malat1* E levels, likely reflected copy number variations of a low abundant and transient unprocessed transcripts.

3. Figure 2: The claim that the two bright nuclear dots represent nascent MALAT1 transcription sites requires experimental support. Confirmation by DNA-FISH or RNA-FISH using probes targeting the 3′ downstream region of MALAT1 (or even the adjacent NEAT1 locus) would strengthen this interpretation.

We thank the Reviewer for suggesting this experiment. As suggested by the Reviewer we performed smRNA-FISH co-detection of *Malat1* and *Neat1* and observed 67% co-localization between the two signals in the nuclei of early G1 cells with cytoplasmic *Malat1* signal (**Fig. 2B**).

4. Figures 3 and 4: Treatments such as ΔA expression, NMD inhibition, and CHX + nocodazole increase cytoplasmic and/or total MALAT1 levels. The authors should exclude the possibility that these effects are secondary to alterations in cell cycle distribution. Assessing cell cycle profiles under each condition using the FUCCI system or established cell cycle markers would strengthen the conclusions.

We agree with the Reviewer that this is a key control. In the revised manuscript, we provide FACS support that KO cells complemented with EV, FL and ΔA have comparable cell cycle profiles (**Fig. S3C**). We also used FACS to show that cells released from mitotic block in the presence of Chx successfully enter G1 at levels comparable to cells released in the absence of a drug (**Fig. S2A**). Lastly, we performed smRNA-FISH on cells released from mitotic arrest in the presence of translation (Chx) or NMD (Smg1 11e) inhibitors and observed that they successfully progress to G1, based on cytological analysis (**Fig. 4F**).

On the other hand – and consistent with the Reviewer's concern - we observed that long-term treatments of asynchronous cells with translation (Chx) or NMD (Smg1 11e) inhibitors prohibited entry into mitosis, thus confounding the interpretation of effects on *Malat1* levels. We, therefore, removed experiments with long-term treatments and focused on short-term inhibition studies post mitotic release.

5. Figures 4B and 4C: Nuclear MALAT1 levels should be assessed to determine whether cytoplasmic MALAT1 can be re-imported into the nucleus, or whether nuclear MALAT1 levels are predominantly determined by newly synthesized transcripts.

We believe that nuclear *Malat1* levels are primarily determined by newly synthesized transcripts, but we also observed that, if *Malat1* is not effectively degraded in early G1, it can be reimported into the nucleus. Below, we have summarized the key data that led us to these conclusions:

In mitotic synchronization and release studies, we observed over 80% loss of *Malat1*, suggesting that the predominant fate of inherited *Malat1* in early G1 is degradation (**Fig. 2F**). Moreover, we performed fractionation studies and observed enrichment of cytoplasmic and depletion of nuclear *Malat1* in cells in early G1 (**Fig. 2E**). Therefore, *Malat1* is degraded in the postmitotic cytoplasm in early G1, while newly transcribed *Malat1* accumulates in the nucleus, consistent with smRNA-FISH observations (**Fig 2A, B; S1H, I**).

To formally address whether inherited cytoplasmic *Malat1* can be imported into the nucleus in the new cell cycle, we rescued *Malat1*-deficient cells with transiently-expressed FL *Malat1* construct. We induced construct expression by treating asynchronous cells with Doxycycline, followed by removal of Doxycycline to cease *Malat1* transcription, treatment with Nocodazole to enrich for mitotic cells, and release into early G1 of the next cell cycle (**Fig. 4D**). To examine the fate of *Malat1* transcribed in the previous cell cycle, we compared cells at

2 and 4 hours post mitotic release to non-released cells. Consistent with our model, by qRT-PCR, we observed 80% and 90% loss of total *Malat1* at 2 and 4 hours post release into the new cell cycle, respectively, compared to non-released cells (**Fig. 4E**). We also quantified in smRNA-FISH images the fraction of cells with cytoplasmic *Malat1* signal as well as the intensity of *Malat1* nuclear signal at the two timepoints post release (**Fig. 4F, G**). At 2 hr post mitotic release, we observed that inherited *Malat1* localized to the cytoplasm in over 30% of the cells, while quantification of nuclear *Malat1* signal revealed over 90% reduction compared to non-released cells, consistent with qRT-PCR analyses (**Fig. 4E-G**). Interestingly, at 4 hr post mitotic release, as the fraction of cells with cytoplasmic *Malat1* dropped below 4%, we observed a small but significant increase in *Malat1* nuclear signal (**Fig. 4F, G**). We concluded that while the majority of inherited *Malat1* is degraded in early G1, the fraction that is not degraded can be reimported into the nucleus. Notably, the amount of nuclear *Malat1* in early G1 appears to be higher in ∆A compared with FL, consistent with incomplete degradation (**Fig. 4B, C**).

Minor comments

1. The Introduction mentions miRNA-mediated regulation as a known cytoplasmic degradation pathway for MALAT1, whereas this study identifies involvement of NMD. The relationship between these pathways should be discussed-whether they are mutually exclusive, act independently, or are context-dependent.

We thank the Reviewer for highlighting this point. Our work highlights the role of NMD in the degradation of *Malat1* in early G1. However, it is possible that, in absence or in addition to NMD, there may be alternative routes for *Malat1* degradation, such as RNAi, as reported. We highlight this possibility in the discussion.

2. In Figure 1D, cells are classified as "Low" or "High" MALAT1 based on nuclear signal intensity, but the threshold used for this classification is not defined. The criteria should be explicitly stated in the Methods or figure legend. Ideally, a histogram of signal intensity distribution should be provided as a supplemental figure to justify the classification.

We thank all three Reviewers for highlighting this omission in our analysis. To strengthen our descriptive conclusions with quantitative analysis, we measured nuclear *Malat1* signal using the Mean Intensity tool in Zen on images acquired with consistent exposure and corrected for background, as described in the revised Methods. This analysis allowed us to compare the distribution and strength of nuclear *Malat1* signal intensity in early vs late passage MEFs (**Fig. 1E**), in early G1 vs G1/S/G2 MEFs (**Fig. 1F, H**) and RPE cells (**Fig. S1F**), and in knockout MEFs rescued with FL vs mutant *Malat1* (**Fig. 4C, G**). We have updated the text to report on the observed statistically significant differences, rather than absolute statements of “low” and “high”.

3. In Figure S1D, the reported difference between early- and late-passage MEFs is difficult to assess from the current images. Wider field-of-view images showing multiple cells simultaneously would better support this claim.

We thank the Reviewer for this request. We have included panoramic images in several figures (**Fig. 1D; S1E, G; 4B, F**). These images illustrate the extent of *Malat1/MALAT1* signal heterogeneity as well as provide representative examples of the frequency of cells with cytoplasmic signal. We have also added quantification of the nuclear signal intensity in early- and late-passage cells (**Fig. 1E**).

4. In Figures 3D, 4B, and 4C, the percentage of cells positive for cytoplasmic MALAT1 is quantified, but the criteria used to define "cytoplasmic MALAT1-positive" cells are not described. The quantification protocol (e.g., signal intensity threshold, localization pattern, nuclear-to-cytoplasmic ratio, background correction) should be clearly stated in the Methods or figure legends. Because this categorical classification is highly dependent on threshold definition and does not necessarily reflect quantitative RNA changes, complementary quantitative

analyses should be included, such as qPCR of cytoplasmic fractions and/or quantification of cytoplasmic MALAT1 signal intensity. In addition, the number of cells analyzed per experiment should be reported to assess the robustness of the data.

These are excellent points, also raised by the other Reviewers, which we have addressed in the revised text and figures and described in Methods:

While image quantification could not be used to directly compare nuclear to cytoplasmic abundance due to the difference in *Malat1* signal area and pattern (condensed nuclear speckles versus diffuse single molecule signals distributed over the cytoplasm), we performed qPCR analysis of fractionated asynchronous and early G1 MEFs (**Fig. 2E**). qRT-PCR analysis allowed direct and quantitative comparison of nuclear and cytoplasmic abundance and revealed patterns, which were consistent with smRNA-FISH analyses.

As suggested by the Reviewer, we also established criteria for defining the threshold for cells that are positive for cytoplasmic *Malat1*. We counted cells as positive if they had 50 or more discrete *Malat1* foci in the cytoplasm. We selected 50 cytoplasmic foci as the threshold to account for the number of foci commonly captured in focus in a single-plane image. This quantification was included in several figures (**Fig. 1F; S1F; SE; and 4F**).

We would like to argue that categorical classification is appropriate in this case, as we observed binary presence or absence of cytoplasmic *Malat1* signal. smRNA-FISH uses multiple probes complementary to transcripts of interest that allows detection of molecules at single molecule resolution. In the nucleus, *Malat1* localizes to speckles, which are bright aggregates of multiple molecules. In the cytoplasm of mitotic and early G1 cells, *Malat1* is visible at single molecule resolution in individual sharp foci that are distinctly above background. The tricky aspect of quantifying *Malat1* in the cytoplasm is that when exposure is set to detect the cytoplasmic portion at single molecule resolution (1000 ms), the nuclear signal is overexposed. We believe that this technical challenge is the main reason why this aspect of *Malat1* biology was not more carefully considered in the past.

5. Cytoplasmic MALAT1 has been reported to interact with Staufen, suggesting a possible contribution of Staufen-mediated decay (SMD). The Discussion should therefore address the potential involvement of SMD, in addition to NMD, in cytoplasmic MALAT1 regulation.

This is a great point, especially since translation and Smg1 are also implicated in SMD. We discuss the possible contribution of SMD to the degradation of cytoplasmic *Malat1*.

6. Statistical analysis: Multiple comparisons are analyzed using t-tests, which is not appropriate. Proper multiple-comparison corrections or alternative statistical methods should be applied. Data variability should be presented as standard deviation (SD) rather than standard error of the mean (SEM), unless a clear rationale for SEM is provided. In several figures (e.g., Figures 2D, 3B, and 4B), statistical significance is not consistently indicated. While not mandatory, including statistical annotations where applicable would improve clarity and consistency.

In the revised version, we have provided details for the appropriate statistical analyses for all experiments. All data have now been updated to include at least three replicates, allowing statistical analyses. We use t-tests to make appropriate paired and unpaired comparisons between paired control and experimental conditions. We confirm that all the data are presented as mean ± SD, rather than SEM.

7. Several abbreviations (e.g., ENE, FUCCI system) are introduced without definition and should be explained

at first mention.

Absolutely! We apologize for the oversight.

Reviewer #3:

In this manuscript, Plasek-Hegde and colleagues investigate mechanisms that regulate Malat1 abundance in cells with different proliferation rates. Using MEFs, they find that cells that proliferate slowly accumulate higher levels of Malat1 than rapidly dividing cells. Cell cycle inhibition and RNA-FISH implicate degradation of cytoplasmic Malat1 upon exit from M phase as a major mechanism responsible for reducing its cellular abundance. Of a series of Malat1 deletion mutants, one lacking a previously identified 5' ORF accumulates to the highest levels and shows increased cytoplasmic localization, suggesting a translation-dependent decay mechanism. To test this, they inhibit NMD using a small molecule NMDi-14 and translation with cycloheximide, both of which rescue RNA abundance in asynchronous cells and in G1. Finally, they show that PC9 cells induced to enter a slow-cycling state express higher levels of MALAT1. This is an interesting manuscript that provides an unexpected mechanism for regulation of a predominantly nuclear "non-coding" RNA. While the available data are highly suggestive, more work is needed to provide fully convincing evidence. In addition, the presentation could be improved in several places by clearer reporting (particularly improving the very sparse figure legends) and improved quantitative methods. Please see below for specific comments:

We thank the Reviewer for the thoughtful summary of our work and for the helpful suggestions how to improve our manuscript. We have addressed each of the specific comments in a point-by-point response below.

1. The authors show that NMD inhibition (Figure 4) and deletion of the 5' end (deltaA; Figure 3) both increase Malat1 abundance, but they do not demonstrate that A) deletion of the 5' end abolishes NMD sensitivity or that B) the wild-type construct and the remaining deletions are NMD-sensitive. In addition, mutants that specifically remove putative ORF M1 should be tested to fully substantiate the authors' claims.

We completely agree with the Reviewer that rescue constructs require characterization. We have performed several additional experiments:

In the revised manuscript, we present data that FL *Malat1* rescue construct is expressed at levels, comparable to endogenous *Malat1*, and degraded in early G1, similar to endogenous *Malat1* (**Fig. 4A; S3D**). Furthermore, we show that this degradation is translation-dependent, similar to endogenous *Malat1* (**Fig. 4A**).

Unexpectedly, we observed that the ΔA construct was also degraded in early G1 in a translation-dependent manner, indicating that lacking region A does not make *Malat1* resistant to degradation (**Fig. 4A, E**). However, we noted that ΔA degradation was less efficient or took longer leading to increased abundance (**Fig. 4A, E; S3D**), prolonged persistence in the cytoplasm (**Fig. 4F**), and reimport into the nucleus (**Fig. 4B, F, G**). Our interpretation of these data is that Region A harbors elements that contribute to but are not required for *Malat1* turnover in early G1. As *Malat1* harbors multiple additional sORFs throughout its length (41 sORFs longer than 20 aa, 55 sORFs longer than 15 aa), one possible explanation is that additional sORFs can trigger NMD.

We also sought to develop a *Malat1* mutant that lacks the initiation codons of several sORFs, including M1. We focused on sORFs located in the 5’ half of *Malat1* (region A and B), where the strongest ribosomal footprints have been reported, and we selected 9 sORFs that also contained strong Kozak sequences. The resulting Δ9ATG *Malat1* mutant had increased abundance compared to FL construct but was significantly less abundant compared to ΔA *Malat1* (**Fig. S3E**). Our conclusion is that the removal of ATGs of a subset of sORFs, including M1, impaired but was not sufficient to abrogate *Malat1* degradation.

Through these studies, we observed that region A, and to a lesser extent 9 sORFs, contribute to *Malat1* turnover, but neither was required for *Malat1* degradation. While the precise mechanism of how *Malat1* engages decay pathways remains unclear, our work reveals that *Malat1* likely harbors multiple redundant elements and provides a path for future hypothesis-based mechanistic dissection.

2. The only evidence provided for NMD involvement is NMDi-14 treatment. One or more additional methods should be provided (e.g. SMG1 inhibitor and/or NMD factor knockdowns) to supplement the use of what remains a fairly poorly characterized drug.

We thank the Reviewer for highlighting the need for an improved approach to investigate the role of NMD, including the suggestion to use of a better characterized SMG1 inhibitor, such as SMG1 11e, compared to the poorly characterized NMDi-14.

We attempted to perform experiments with (1) NMD inhibitors, including NMDi-14 and Smg1 11e and (2) NMD factor RNAi knockdown, focusing on the decapping enzyme Dcp2, since *Malat1* lacks a polyadenylation tail and is considered to be an unspliced transcript.

One of our challenges was that long-term inhibition of translation or NMD in asynchronous cells prevented entry into mitosis, limiting our investigation into the effects of these drugs on postmitotic *Malat1* turnover. To overcome this challenge, we performed mitotic synchronization and release experiments in the absence or presence of drugs (**Fig. 3A**). In the presence of Chx or Smg1 11e, we observed a 2-fold increase in *Malat1* abundance (**Fig. 3B, D**) and retention of cytoplasmic *Malat1* in >90% of cells compared to 10% in control cells (**Fig. 3E**) at 2 hours post mitotic release. These robust results, reported in **Figure 3**, led to the conclusion that translation and Smg1 activity are required for postmitotic *Malat1* degradation, consistent with NMD.

We would like to note that NMDi-14 treatment in mitotically released cells did not have the same effects as Smg1 inhibition, described above. We became concerned that our previous observation for accumulation of cytoplasmic *Malat1* in cells treated with NMDi-14 for 24 h may have been due to nonspecific effects. Indeed, 24 h treatment with Smg1 11e led to interphase arrest and lack of progression through mitosis. We thank the Reviewer for highlighting the problems with NMDi-14 and suggesting Smg1 inhibitors.

We also tested 5 independent *Dcp2*-specific siRNAs (individually or in combination) and achieved 40-60% knockdown. We focused on the decapping factor Dcp2 because *Malat1* lacks a polyA tail. *Dcp2* knockdown led to a 1.5-fold increase in *Malat1* levels in asynchronous cells (n=6 biological replicates, $p<0.05$), consistent with our model. However, due to concerns that long-term *Dcp2* knockdown may alter cell cycle distribution, we did not in include these data in the revised manuscript.

In addition, it is important to show that decay is actually occurring under normal conditions--this should be done with 4SU or other metabolic labeling-based approaches (such as Roadblock-PCR), not with Actinomycin D treatment, which causes profound cellular stress.

This is another excellent point. We sought to reconcile the published long half-life of *Malat1* (>16 hr) with our model that *Malat1* is degraded at the onset of each cell cycle. For this, we treated cells with EU to label nascent *Malat1* and then measured the decay of EU-labeled *Malat1* over time. This approach revealed a half-life of approximately 6.5 hours, consistent with a cell cycle length of 13 hours in p53-deficient MEFs (**Fig. 2G).**

3. A previous study (PMID: 33784500) showed that a truncated, polyadenylated isoform of Malat1 can be produced under certain conditions. The qPCR approach taken by the authors cannot distinguish among

different potential Malat1 isoforms that might be produced upon cellular perturbations or upon mutation of reporter constructs. Northern blotting or sequencing-based methods capable of detecting the full array of Malat1 isoforms expressed from the endogenous locus and from the reporter constructs are needed.

To address this excellent point, we performed qPCR analysis with primers, specific to regions A through D, spanning the entire length of the processed transcript in several key experiment (**Fig. 1A**). These data, included in supplementary figures (**Fig. S1C, S2B, S3D**), are consistent with full length *Malat1* being produced in all cases.

Also related to this point, I was not able to discern the PCR primers used for the quantification of RNA levels in Figure 3B. The primers listed in the supplementary table both recognize regions of Malat1 that are absent in at least one deletion construct. Please clarify and ensure that the methods used are capable of detecting all mutants equally.

We assure the Reviewer that we used appropriate primers to detect full length and deletion mutants of *Malat1*. The initial figure was misleading as it was a mix of two sets of primers. We have corrected this in **Fig. S3D** and included the sequence of appropriate primers in Supplementary Table. We thank the Reviewer for bringing this up.

4. Also in Figure 3B, the experiments are performed in asynchronous cells, but the magnitude of the effect of the deltaA deletion greatly exceeds the effects observed in any of the experiments involving the endogenous Malat1. Can the authors comment on why this might be the case?

The Reviewer is referring to Fig. 3B, corresponding to **Fig. 4A, E and S3D** in the revised version, which show that ∆A *Malat1* is significantly more abundant compared to endogenous or FL *Malat1*. We believe that the accumulation of *Malat1* in asynchronous cells is the consequence of incomplete degradation of cytoplasmic ∆A *Malat1* in early G1, followed by reimport of undegraded ∆A *Malat1* into the nucleus (**Fig. 4D-G**). It is also possible that there may be additional factors promoting ∆A *Malat1* abundance.

5. I'm surprised by the small decreases observed in "nascent" Malat1 levels in M phase (Figure 1E, Figure 2D, 4D). There should not be ongoing transcription in M phase, so it seems that the RNA detected may be unprocessed rather than nascent or that there may be a problem with the qPCR assay used (it can be difficult to accurately quantify low-abundance downstream products). In addition, it is quite unexpected that the levels of "nascent" RNA would significantly increase with cycloheximide treatment (Figure 4D). Can the authors explain this observation?

These are very thoughtful observations, also brought up by other Reviewers. We believe that the observed differences were misleading. We find that transcripts detected with primer sets in region E are several hundred-fold less abundant compared to transcripts detected with primer sets in regions A-D, indicating efficient processing of nascent transcript. We have now plotted relative rather than normalized levels on the same graphs, which clearly shows that processed *Malat1* transcripts are the predominant species **(Fig. 1B, C; 2F**). We believe that the inconsistent fold change differences we previously observed, when plotting normalized *Malat1* levels, were not functionally significant but reflected copy number variations of low abundant and transient unprocessed transcripts.

6. In several places, quantification and methods reporting are lacking:

We thank all three Reviewers for highlighting this omission in our analysis. To strengthen our descriptive conclusions, we have now performed detailed quantitative analysis, described below:

a. Figure 1D: What defines "high" and "low" signal? It would be much better to show distributions of signal intensities rather than taking this binning approach, which seems to exaggerate the magnitude of the differences across the cell cycle.
b. Figure S1D: Quantification is needed to substantiate the claim in the text that early-passage cells show more variability in nuclear RNA abundance than late-passage cells.

We measured **nuclear *Malat1* signal** using the Mean Intensity tool in Zen on images acquired with consistent exposure and corrected for background, as described in the revised Methods. This analysis allowed us to compare the distribution and strength of nuclear *Malat1* signal intensity in early vs late passage MEFs (**Fig. 1E**), in early G1 vs G1/S/G2 MEFs (**Fig. 1F, H**) and RPE cells (**Fig. S1F**), and in knockout MEFs rescued with FL vs mutant *Malat1* (**Fig. 4C, G**). We now provide quantitative analysis, rather than subjective "high/low" binning.

c. Figure 4B and C: How was the cutoff drawn to determine cells with positive/negative cytoplasmic signal? Again, a distribution of intensities would be preferable here.

We quantified **the fraction of cells with cytoplasmic *Malat1*** by counting cells as positive if they had 50 or more discrete single molecule *Malat1* foci in the cytoplasm. This categorical classification was appropriate as the presence or absence of *Malat1* in the cytoplasm of WT MEFs or RPE cells was a largely binary occurrence with cells either lacking cytoplasmic foci or harboring hundreds of discrete cytoplasmic foci. The cells with cytoplasmic *Malat1* signal were consistently cytologically identifiable as being in the early G1 stage of the cell cycle, based on the proximity of daughter nuclei. We selected 50 cytoplasmic foci as the threshold to account for the number of foci commonly captured in focus in a single-plane image. This quantification has been included in several figures (**Fig. 1F, S1F, 3E, 4F**).

It is important to note that smRNA-FISH uses multiple probes complementary to transcripts of interest that allows detection of molecules at single molecule resolution. In the nucleus, *Malat1* localizes to speckles, which are bright aggregates of multiple molecules. In the cytoplasm of mitotic and early G1 cells, *Malat1* is visible at single molecule resolution in individual sharp foci that are distinctly above background. The tricky aspect of quantifying *Malat1* in the cytoplasm is that when exposure is set to detect the cytoplasmic portion at single molecule resolution (1000 ms), the nuclear signal is overexposed. We believe that this technical challenge is the main reason why this aspect of *Malat1* biology was not more carefully considered in the past.

d. Throughout, figure legends require reporting of exact replicate numbers, numbers of cells counted per replicate, and methods used to determine error bars and p-values. SD rather than SEM should be used.

We have included these data by improving figure legends, specifying number of cells, replicates, and statistical methods. We have plotted mean ± SD. We apologize for using the wrong method.

e. Figure 3C, the authors state that they "observed [sic] high rate of co-occurrence of abundant nuclear and cytoplasmic Malat1 staining...," but no quantification is provided to substantiate this claim.

In new **Fig. 4B, C**, we used quantification of mean intensity to compare nuclear signal in ∆A and FL cells and reported on the statistical difference between the two samples.

Minor points:

1. The idea that NMD takes place in the pioneer round of translation is controversial and should not be stated as fact (see for example: PMIDs: 23665581, 23665580, 31155380).

This is a very helpful comment. We have removed this controversial statement from our discussion. We thank the Reviewer for sharing with us relevant manuscripts.

2. Please ensure original discovery papers are cited alongside recent reviews in all sections (e.g., FUCCI, smFISH/IF methods, NMD models) and add missing primary references where appropriate.

We have done our best to include appropriate primary references.

3. The authors write that there "were no consistent changes in nascent Malat1 (Figure 1E)," but the figure shows significant differences among cycling, M-phase, and G2 phase conditions.

We agree that this part of the manuscript was very confusing, as highlighted by all three Reviewers. We have updated our statement to focus on the relative abundance of the predominant processed *Malat1* transcripts.

4. Figure 2A shows FISH of fixed cells, so it's not clear to me how the authors determine that the Malat1 signal "abruptly disappeared."

We agree with the Reviewer's concern about our word choice. The concept of "abrupt" disappearance was based on the observation that we only see *Malat1* in the cytoplasm in approximately 10% of cells that are cytologically in the earliest stages of G1, with lack of notable cytoplasmic *Malat1* in other stage of interphase. However, we agree with the Reviewer that the use of a kinetic word to describe a static observation is not appropriate. We have now used data from **Fig. 2E, 4F** to state that *Malat1* cytoplasmic signal disappears within the first 2-4 hours of the cell cycle.

July 13, 2026

RE: JCB Manuscript #202512059R

Nadya Dimitrova
Yale University

Dear Dr. Dimitrova:

Thank you for submitting your revised manuscript entitled "Cell cycle-dependent translation-mediated turnover of the long noncoding RNA Malat1". The reviewers all now support publication so we would be happy to publish your paper in JCB pending final revisions necessary to meet our formatting guidelines (see details below).

**In your final revision, please be sure to address reviewer #2's final minor concerns.

To avoid unnecessary delays in the acceptance and publication of your paper, please read the following information carefully.

A. MANUSCRIPT ORGANIZATION AND FORMATTING:

Full guidelines are available on our Instructions for Authors page, http://jcb.rupress.org/submission-guidelines#revised.
Submission of a paper that does not conform to JCB guidelines will delay the acceptance of your manuscript.

**1) Text limits: Character count for Reports is < 20,000, not including spaces. Count includes title page, abstract, introduction, the joint Results & Discussion, and acknowledgments. Count does not include materials and methods, figure legends, references, tables, or supplemental legends.

**Please note that Results & Discussion are supposed to be a joint section in the JCB Report format. We kindly ask you to combine the Results and Discussion in your paper.

2) Figures limits: Reports may have up to 5 main text figures.

**3) Figure formatting:
**Scale bars must be present on all microscopy images, including inset magnifications.
Molecular weight or nucleic acid size markers must be included on all gel electrophoresis. Aspect ratios of images may not be altered.

4) Statistical analysis: Error bars on graphic representations of numerical data must be clearly described in the figure legend. The number of independent data points (n) represented in a graph must be indicated in the legend. Statistical methods should be explained in full in the materials and methods. For figures presenting pooled data the statistical measure should be defined in the figure legends. Please also be sure to indicate the statistical tests used in each of your experiments (either in the figure legend itself or in a separate methods section) as well as the parameters of the test (for example, if you ran a t-test, please indicate if it was one- or two-sided, etc.). Also, if you used parametric tests, please indicate if the data distribution was tested for normality (and if so, how). If not, you must state something to the effect that "Data distribution was assumed to be normal but this was not formally tested."

**5) Abstract and title:
**The abstract should be no longer than 160 words and should communicate the significance of the paper for a general audience.
The title should be less than 100 characters including spaces. Make the title concise but accessible to a general readership.

6) Materials and methods: Should be comprehensive and not simply reference a previous publication for details on how an experiment was performed. Please provide full descriptions in the text for readers who may not have access to referenced manuscripts.

7) All antibodies, cell lines, animals, and tools used in the manuscript should be described in full, including accession numbers for materials available in a public repository such as the Resource Identification Portal. Please be sure to provide the sequences for all of your primers/oligos and RNAi constructs in the materials and methods. You must also indicate in the methods the source, species, and catalog numbers (where appropriate) for all of your antibodies. Please also indicate the acquisition and quantification methods for immunoblotting/western blots.

8) Microscope image acquisition: The following information must be provided about the acquisition and processing of images:
a. Make and model of microscope

b. Type, magnification, and numerical aperture of the objective lenses
c. Temperature
d. Imaging medium
e. Fluorochromes
f. Camera make and model
g. Acquisition software
h. Any software used for image processing subsequent to data acquisition. Please include details and types of operations involved (e.g., type of deconvolution, 3D reconstitutions, surface or volume rendering, gamma adjustments, etc.).

9) References: There is no limit to the number of references cited in a manuscript. References should be cited parenthetically in the text by author and year of publication. Abbreviate the names of journals according to PubMed.

10) Supplemental materials: There are strict limits on the allowable amount of supplemental data. Reports may have up to 3 supplemental figures. Please also note that tables, like figures, should be provided as individual, editable files. A summary of all supplemental material should appear at the end of the Materials and methods section.

11) eTOC summary: A ~40-50-word summary that describes the context and significance of the findings for a general readership should be included on the title page. The statement should be written in the present tense and refer to the work in the third person.

12) Conflict of interest statement: JCB requires inclusion of a statement in the acknowledgements regarding competing financial interests. If no competing financial interests exist, please include the following statement: "The authors declare no competing financial interests." If competing interests are declared, please follow your statement of these competing interests with the following statement: "The authors declare no further competing financial interests."

13) ORCID IDs: ORCID IDs are unique identifiers allowing researchers to create a record of their various scholarly contributions in a single place. Please note that ORCID IDs are now *required* for all authors. At resubmission of your final files, please be sure to provide your ORCID ID and those of all co-authors.

14) A separate author contribution section following the Acknowledgments. All authors should be mentioned and designated by their full names. We encourage use of the CRediT nomenclature.

Please note that JCB now requires authors to submit Source Data used to generate figures containing gels and Western blots with all revised manuscripts. This Source Data consists of fully uncropped and unprocessed images for each gel/blot displayed in the main and supplemental figures. For assays performed using capillary electrophoresis and/or immunoassay-based detection, authors should instead provide the electropherogram graph(s) for each experiment, plotting fluorescence/chemiluminescence intensity vs. molecular weight/size. Please be sure to provide one Source Data file for each figure gels, blots, and/or capillary electrophoresis assays along with your revised manuscript files. File names for Source Data figures should be alphanumeric without any spaces or special characters (i.e., SourceDataF#, where F# refers to the associated main figure number or SourceDataFS# for those associated with Supplementary figures). For traditional gels and blots, the lanes of the gels/blots should be labeled as they are in the associated figure, the place where cropping was applied should be marked (with a box), and molecular weight/size standards should be labeled wherever possible. For capillary electrophoresis assays, each trace in the graph should be color-coded and labeled to indicate which protein, gene, or sample is being measured (please try to avoid red/green combinations to accommodate our color-blind readers).

Source Data files will be made available to reviewers during evaluation of revised manuscripts and, if your paper is eventually published in JCB, the files will be directly linked to specific figures in the published article.

Source Data Figures should be provided as individual PDF files (one file per figure). Authors should endeavor to retain a minimum resolution of 300 dpi or pixels per inch. Please review our instructions for export from Photoshop, Illustrator, and PowerPoint here: https://rupress.org/jcb/pages/submission-guidelines#revised

Journal of Cell Biology now requires a data availability statement for all research article submissions. These statements will be published in the article directly above the Acknowledgments. The statement should address all data underlying the research presented in the manuscript. Please visit the JCB instructions for authors for guidelines and examples of statements at (https://rupress.org/jcb/pages/editorial-policies#data-availability-statement).

B. FINAL FILES:

Please upload the following materials to our online submission system. These items are required prior to acceptance. If you have any questions, contact JCB's Managing Editor, Lindsey Hollander (lhollander@rockefeller.edu).

-- An editable version of the final text (.DOC or .DOCX) is needed for copyediting (no PDFs).

-- High-resolution figure and MP4 video files: See our detailed guidelines for preparing your production-ready images, https://jcb.rupress.org/fig-vid-guidelines.

-- Cover images: If you have any striking images related to this story, we would be happy to consider them for inclusion on the journal cover. Submitted images may also be chosen for highlighting on the journal table of contents or JCB homepage carousel. Images should be uploaded as TIFF or EPS files and must be at least 300 dpi resolution.

It is JCB policy that if requested, original data images must be made available to the editors. Failure to provide original images upon request will result in unavoidable delays in publication. Please ensure that you have access to all original data images prior to final submission.

The license to publish form must be signed before your manuscript can be sent to production. A link to the license to publish form will be sent to the corresponding author only. Please take a moment to check your funder requirements before choosing the appropriate license.

Additionally, JCB encourages authors to submit a short video summary of their work. These videos are intended to convey the main messages of the study to a non-specialist, scientific audience. Think of them as an extended version of your abstract, or a short poster presentation. We encourage first authors to present the results to increase their visibility. The videos will be shared on social media to promote your work. For more detailed guidelines and tips on preparing your video, please visit https://rupress.org/jcb/pages/submission-guidelines#videoSummaries.

Thank you for your attention to these final processing requirements. Please revise and format the manuscript and upload materials within 14 days. If you need an extension for whatever reason, please let us know and we can work with you to determine a suitable revision period.

Please contact the journal office with any questions at cellbio@rockefeller.edu.

Thank you for this interesting contribution, we look forward to publishing your paper in Journal of Cell Biology.

Sincerely,

Jens Lykke-Andersen
Monitoring Editor
Journal of Cell Biology

Gabriele Stephan
Scientific Editor
Journal of Cell Biology

Reviewer #1:

The authors have added additional new data and analyses that addressed my prior concerns.

Reviewer #2:

The reviewer recognizes that the authors have adequately addressed all previously raised concerns. Although this point does not critically affect the overall conclusions, the relative abundance of unprocessed versus processed MALAT1 cannot be accurately compared using RT-PCR with different primer sets, as amplification efficiencies are likely to differ between them. Therefore, the authors' claim that unprocessed transcripts account for less than 1% of total transcripts (Figure 1B and 1C; page 4, second line from the bottom; and related to major comments #1 and #2) is not sufficiently supported by the presented data. Absolute quantification using a standard DNA template or an equivalent approach would be required to substantiate this conclusion. Once this issue is addressed, the manuscript would be suitable for publication in the Reports of the Journal of Cell Biology.

Reviewer #3:

The authors have comprehensively responded to my previous comments. I continue to think that this is a fascinating story with interesting implications for MALAT1 and NMD.
