# Supplementary material for: Cell cycle–dependent translation-mediated turnover of the long noncoding RNA Malat1
Source: J Cell Biol. 2026 Aug 25;225(10):e202512059. doi: 10.1083/jcb.202512059 (PMC13505482; doi:10.1083/jcb.202512059)
Supplement: Supplementary file 2 [file jcb_202512059_sourcedataf2.pdf]

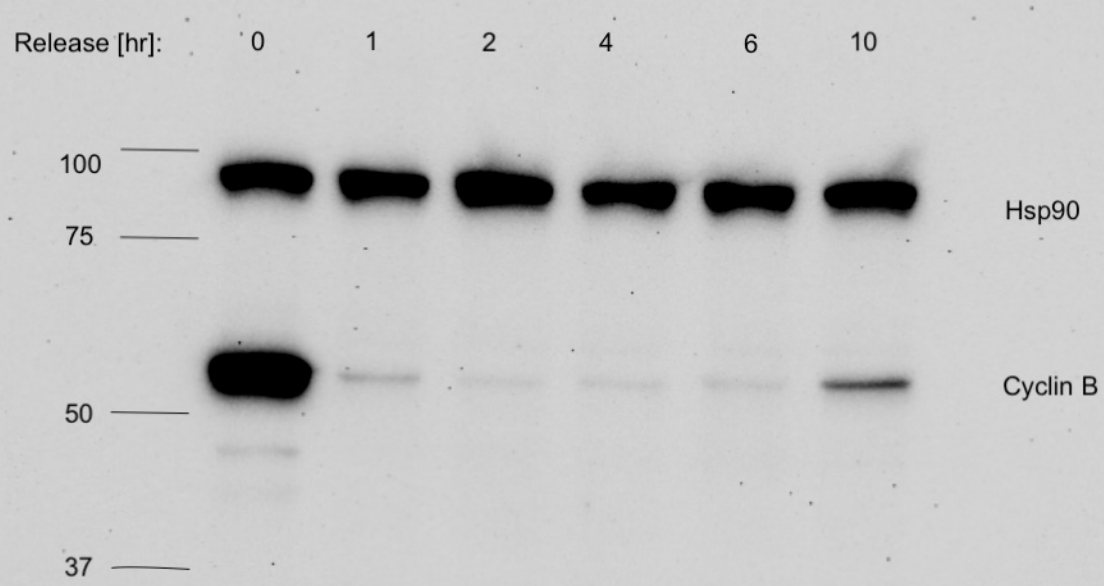
Release [hr]:
0
1
2
4
6
10
100
75
50
37
Hsp90
Cyclin B
